# Quantifying the metabolic heat from aerobic methanotrophs

**DOI:** 10.1128/mbio.00980-26

**Published:** 2026-09-18

**Authors:** Tymon A. Herzyk, Tania L. Gomez-Borraz, Rebeca Gonzalez-Cabaleiro, William T. Sloan

**Affiliations:** 1 James Watt School of Engineering, University of Glasgow 3526, Glasgow, Scotland; 2 Delft University of Technology 2860, Delft, the Netherlands; Shanghai Jiao Tong University, Shanghai, China

**Keywords:** methane, methanotrophs, metabolic heat

## Abstract

Microorganisms must dissipate some energy as heat to keep energy-capturing reactions thermodynamically favorable. For methane-oxidizing bacteria (methanotrophs), circumstantial evidence, such as temperature increases within biofilters and landfills, suggests that the metabolic heat they generate might be valorized. However, the precise rate and yield of heat produced by different methanotrophic species have not been measured. Here, we measured heat flux from pure cultures of two methanotrophs, *Methylomonas methanica* S1 (type I) and *Methylosinus trichosporium* OB3b (type II). Isothermal calorimetry of sealed batch cultures at 30°C under methane concentrations of 8%, 4%, and 2% (vol/vol) in air showed that both species dissipated approximately 60% of the substrate energy as heat. Biomass-specific heat yields ranged from 1,473 to 2,000 kJ/Cmol for *M. methanica* S1 and from 2,638 to 3,728 kJ/C-mol for *M. trichosporium* OB3b. These values exceed experimentally reported heat yields for other aerobic microorganisms, suggesting that methane-oxidizing bacteria may represent the upper limit of metabolic heat production among aerobic microorganisms and are unique in their ability to dissipate significant metabolic heat at comparative biomass yields. This work provides the first direct quantification of heat generation during biological methane oxidation, establishing a necessary foundation to leverage this resource within methane mitigation biotechnologies and wider engineering applications.

**IMPORTANCE:**

Methanotrophic bacteria dissipate substantial metabolic energy as heat while oxidizing methane, a trait that may have evolved to manage excess energy. If harnessed, this process positions these microorganisms as effective agents for passively mitigating methane emissions while providing an alternative source for energy recovery. Although field studies have suggested the potential for significant heat generation from biological methane oxidation, experimental quantification has been lacking. Here, we quantitatively measured the heat evolved from two species of methanotrophs across different methane concentrations. The observed heat yields are far higher than previously recorded for aerobic microorganisms, setting a new empirical boundary for aerobic metabolic heat production.

## INTRODUCTION

Schrödinger famously stated that “an organism feeds upon negative entropy” (1), succinctly capturing the constraints imposed on living systems by the second law of thermodynamics. This principle has motivated subsequent theoretical descriptions of biological growth grounded in macroscopic chemical energetics (2–7). Historically, calorimetric measurements of the heat flux from microorganisms have proved useful in validating these models (8–19). More recently, thermodynamic studies have focused on analyses at the level of metabolic networks and pathways (20–24). However, a recent meta-analysis of historical heat-flux data sets underscores the continued relevance of calorimetric approaches while also highlighting a notable lack of such measurements for methane-oxidizing bacteria (25).

Methane’s status as the most reduced form of carbon makes its complete oxidation highly exothermic (Δ_*c*_H° = −890.71 kJ/mol) (26). This wealth of per-carbon energy places methane-oxidizing bacteria at the extreme of the metabolic versatility exhibited by unicellular organisms. Therefore, methanotrophic bacteria are proposed to have the potential to produce significant heat yields (3, 6, 8, 27, 28). While observed temperature increases of approximately 40°C within methanotrophic biofilters provide circumstantial evidence for this (29, 30), the exact quantity of heat produced by methanotrophic bacteria has not been experimentally quantified. Our aim is to remedy this.

Aerobic methanotrophs fall under two phyla, Proteobacteria, split into classes of Alphaproteobacteria and Gammaproteobacteria, and Verrucomicrobia (31, 32). It is more common to categorize them into simplified functional types, type I, type II, and type X, which reflect differences in physiological taxonomy, chemotaxonomy, phylogeny, and biochemical aspects (33). Indeed, while the capacity to consume methane to generate energy, biomass, and carbon dioxide is common to all methanotrophs (31, 34), the biochemistry of these steps can differ (34, 35), leading to versatility and the ability of methanotrophs to occupy different ecological niches (36–38).

The catabolic pathway employed by methanotrophs enables the complete oxidation of methane to carbon dioxide, through the intermediates methanol, formaldehyde, and formate, at ambient conditions (31)—a process that eludes current technology (39). Formaldehyde and formate serve as the entry point for the ribulose monophosphate (RuMP) and serine cycles used by type I and type II methanotrophs, respectively, for carbon assimilation (34). Certain methanotrophs have also been shown to express ribulose-bisphosphate carboxylase, enabling the fixation of carbon dioxide through operation of the Calvin-Benson-Bassham cycle (34, 40, 41).

The ability of methanotrophs to fully oxidize methane under ambient conditions is not only biochemically remarkable but also environmentally significant. Methane is the second most abundant greenhouse gas in the atmosphere and holds a global warming potential of 82.5 ± 25.8 and 79.7 ± 25.8 times that of carbon dioxide over a 20-year period, when emitted from fossil fuels or non-fossil sources, respectively (42). As a result, reducing methane emissions from, for example, waste and wastewater technologies—a leading anthropogenic source of methane (43)—has been identified as a feasible opportunity for scaling up climate action (44). This presents opportunities to exploit the metabolic pathways exhibited by methanotrophs to improve the sustainability of biotechnologies (45).

In many engineered waste treatment systems with methane-rich gas, including biogas from anaerobic digestion of organic waste, burning methane in combined heat and power plants adds value to the technology (46, 47). However, many wastewater and waste systems are decentralized, passive, and small-scale, where gaseous methane concentrations are too low to burn and therefore are emitted directly into the environment (48–50). Although these systems are individually small, their collective emissions are considerable. For example, while only 16% of wastewater in the United States is treated in septic systems, it has been suggested that these systems contribute 44% of the 485 kt of total methane emissions from all wastewater treatment (51). Gupta et al. (52) show that the per capita carbon footprint of rural wastewater treatment in Scotland is seven times that of centralized urban treatment, and most of this can be attributed to methane emissions from septage. These systems are much neglected, and as a result, they are becoming increasingly prone to failure (53, 54). One of the key challenges is the lack of standardized regulation, placing the responsibility and cost of these systems on individual homeowners (55–58). The likelihood of individuals augmenting their system is therefore increased if some additional economic or resource benefit can be accrued.

Biological removal of methane, through the use of methanotrophs, is particularly attractive for systems with low methane concentrations (59–63). Biofilters containing methanotrophs have been deployed to reduce methane emissions from landfills (64–67) and animal husbandry (68–71), and while biological methane oxidation of wastewater is known to occur (72), the proposal of incorporating biofilters within decentralized wastewater technologies, like septic tanks, is only a recent development (73–75). The use of aerobic methanotrophs also lends itself to more circular economy approaches. A review by Strong et al. (76) highlights that methanotrophs can be used to generate a wide range of useful products; however, the generation of such products requires highly controlled facilities and is therefore likely unsuitable for small-scale decentralized wastewater systems. Our interest is in exploiting the thermodynamic properties of these organisms to enhance the economic viability of mitigation technologies through the capture of low-grade metabolic heat.

In lieu of direct measurements of methanotrophic heat production, theoretical approaches are used (30, 77–79). Within these models, the biological oxidation of methane is often depicted as:


$${CH}_{4}+\left(2-x\right)O_{2}\to \left(1-x\right){CO}_{2}+\left(2-x\right)H_{2}O+xC_{biomass}$$

where *x* is the stoichiometric coefficient relating the proportion of carbon used for biomass. This is often assumed to be 0.5 (30, 77, 80, 81) and does not account for variation as a result of environmental conditions (82) or metabolic differences (83, 84). Theoretical approaches have only been tenuously linked to experimental evidence. Thus, if we are to rationally design biotechnologies to generate heat from biological methane oxidation, then it is imperative that we empirically quantify the heat dissipated by methanotrophs and validate, or update, theoretical models for their energetics.

In this study, we have developed a robust approach to characterize the heat dissipated by different species of methanotrophs. Pure cultures of *Methylomonas methanica* S1 and *Methylosinus trichosporium* OB3b were cultivated separately within a Calimetrix I-CAL HPC 4000 Isothermal Calorimeter, allowing us to measure the heat dissipation *in situ*. This was done for three distinct concentrations of methane (8%, 4%, and 2% [vol/vol] in air), which represent typical low-concentration methane in air streams while avoiding oxygen limitation (75, 85). For cultures within the calorimeter, measurements of environmental and growth parameters, such as optical density, pH, headspace composition, and pressure, were taken at the start and end of each growth period. To reproduce how these values change over time, external cultures were grown in parallel to those within the calorimeter and sampled routinely over the entire growth period.

## RESULTS AND DISCUSSION

### Growth kinetics

Table 1 gives a summary of the differences between the growth of *M. methanica* S1 and *M. trichosporium* OB3b. It compares the growth yield, *Y_X_*_/_*_S_*; the specific growth rate, μ; the percentage of substrate carbon assimilated into biomass, %biomass; the percentage of substrate carbon fully oxidized to carbon dioxide, %CO_2_; and the ratio between the end use of substrate carbon. A ratio >1 indicates that a larger proportion of the substrate carbon consumed is oxidized to carbon dioxide rather than assimilated into biomass. These quantities are calculated per culture, and the values presented in Table 1 are the means from the biological triplicate cultures for each of the concentrations of methane (8%, 4%, and 2% [vol/vol] in air). Yields obtained from external cultures and calorimeter cultures are presented separately and reported in both g/g and Cmol/mol. For the substrate, mol and Cmol are equivalent as methane contains only a single carbon atom. Specific growth rates (µ) could only be calculated from external cultures due to the lack of biomass time series from calorimeter cultures.

**TABLE 1:** Final yields of cell dry weight (*Y_X_*_/*S*_), specific growth rates (µ),^*a*^ percentage of consumed carbon converted to biomass (%biomass) and carbon dioxide (%CO_2_), and the carbon partitioning ratio between biomass and carbon dioxide for pure batch cultures of *M. methanica* S1 (M.m) and *M. trichosporium* OB3b (M.t) grown under three concentrations of methane (8%, 4%, and 2% [vol/vol] in air)^*b*^

Species	Exp		*Y_X_*_/*S*_				µ (h^−1^)		%biomass		%CO_2_		Ratio (–)	
	Cult	Methane	g/g		Cmol/mol									
			Mean	SD	Mean	SD	Mean	SD	Mean	SD	Mean	SD	Mean	SD
*M. m*	Cal	8%	0.53	0.00	0.33	0.00	–	–	33.45	0.08	58.84	2.88	1.76	0.08
		4%	0.43	0.01	0.27	0.01	–	–	27.19	0.71	57.75	1.28	2.13	0.10
		2%	0.56	0.01	0.35	0.01	–	–	35.04	0.68	51.08	3.86	1.46	0.08
*M. t*	Cal	8%	0.25	0.01	0.15	0.01	–	–	15.41	0.62	66.26	0.48	4.31	0.19
		4%	0.22	0.01	0.14	0.00	–	–	13.86	0.38	64.42	1.04	4.65	0.11
		2%	0.30	0.03	0.19	0.02	–	–	19.14	1.66	63.84	1.21	3.35	0.31
*M. m*	Ext	8%	0.55	0.00	0.34	0.00	0.061	0.001	34.31	0.09	57.96	0.84	1.69	0.03
		4%	0.47	0.06	0.30	0.04	0.062	0.002	29.64	3.57	56.21	2.33	1.92	0.33
		2%	0.55	0.04	0.34	0.02	0.059	0.002	34.32	2.35	55.35	1.90	1.62	0.12
*M. t*	Ext	8%	0.26	0.00	0.16	0.00	0.016	0.006	16.16	0.16	70.42	1.49	4.36	0.06
		4%	0.27	0.03	0.17	0.02	0.007	0.002	16.94	1.61	71.27	2.73	4.22	0.27
		2%	0.36	0.05	0.22	0.03	0.007	0.002	22.40	3.44	68.48	7.85	3.08	0.35

^*a*^ “–” indicates that specific growth rates are only calculated for externally grown cultures due to lack of biomass timeseries from cultures grown within the calorimeter.

^*b*^ Cult, culture. “Cal*”* refers to cultures grown within the calorimeter, while “Ext” refers to externally grown cultures. Means are calculated from biological triplicates, with standard deviations shown.

### Growth rates and yields

*M. methanica* S1 was found to have a higher specific growth rate than *M. trichosporium* OB3b for all three concentrations of methane. The growth rates of both species are low in comparison to those reported elsewhere, which range from 0.005 to 0.4 h^−1^ (86) and are known to depend on multiple factors (37, 87–92). Here, no agitation of cultures occurred, which limits the gas-liquid transfer to passive diffusion. Given that methane has low solubility, one would expect a decreased availability of substrate compared to stirred cultures. Indeed, reducing shaking from 300 to 130 rpm has been found to result in a 46% reduction in the growth rate of *Methylomonas albus* BG8 (type I methanotroph), and it is believed that an rpm of less than 240 acts to instill mass transfer-limited growth (37). The reduced growth rates measured here for *M. trichosporium* OB3b are likely attributable to carbon dioxide scarcity. Previous studies have shown that ambient carbon dioxide concentrations limit both the rate of growth of type II methanotrophs and the production of total organic carbon (87, 93). This is a consequence of the requirement of carbon dioxide during the conversion of phosphoenol pyruvate to oxaloacetate within the serine cycle utilized by type II methanotrophs (87).

*M. methanica* S1 was also found to have higher growth yields than *M. trichosporium* OB3b for all methane concentrations (Table 1). The ATP requirements for anabolism in *M. trichosporium* OB3b (serine pathway) are higher than those for *M. methanica* S1 (RuMP pathway), which is grounds for expecting higher biomass yields for *M. methanica* S1 (83). The higher growth rates and yields observed for *M. methanica* S1 compared to *M. trichosporium* OB3b are consistent with observations that type I outcompete type II methanotrophs in methane-limiting environments (36, 37, 94, 95).

### Carbon partitioning

**Fig 1:**
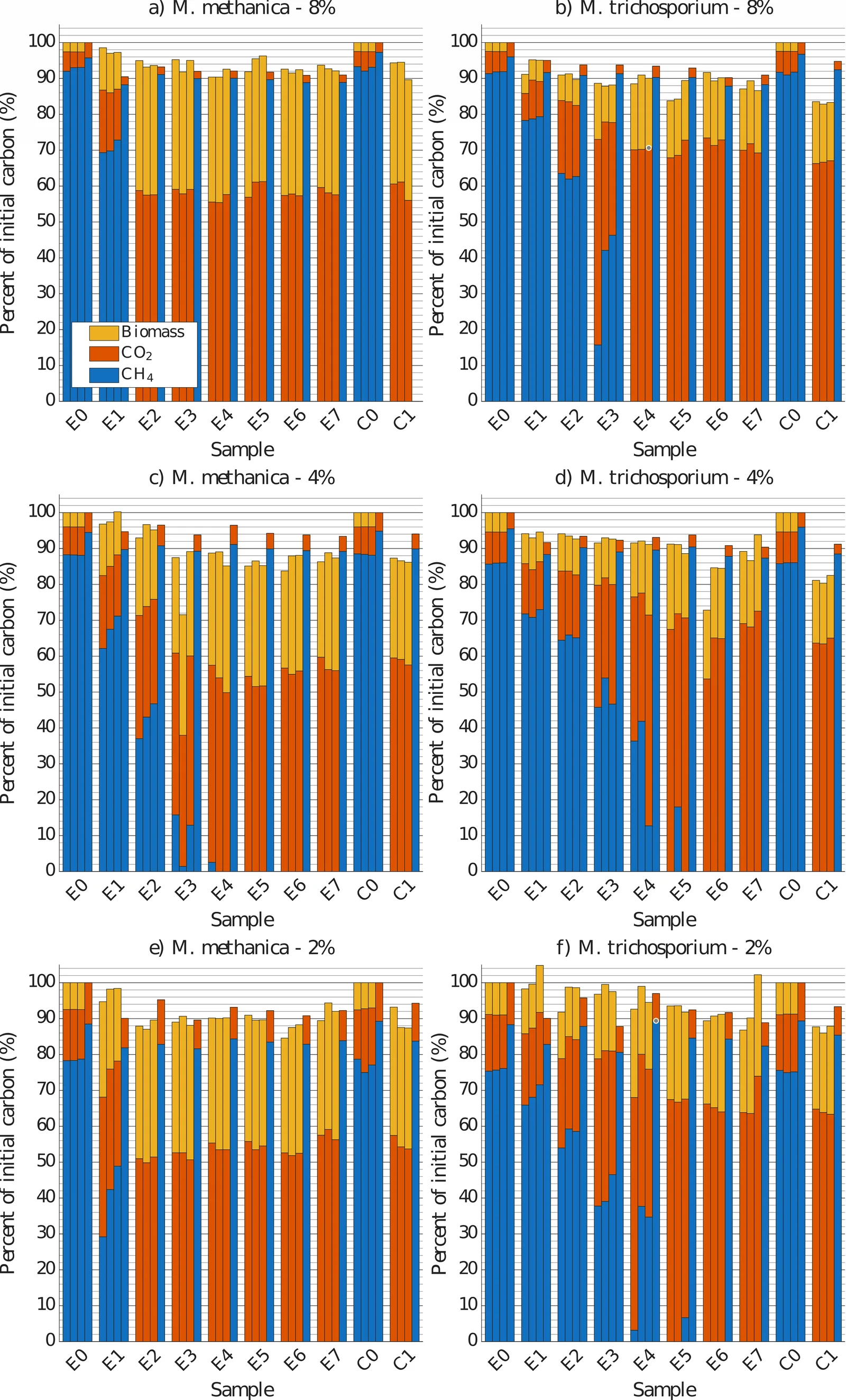
Carbon distribution expressed as a percent of the initial carbon within the system. Data are from batch cultures of *M. methanica* S1 and *M. trichosporium* OB3b grown under three methane concentrations (8%, 4%, and 2% [vol/vol] in air). Samples E0*–*E7 are external cultures; C0*–*C1 are calorimeter cultures. Exact time each sample was taken is shown in Fig. S1 and S2. Columns 1*–*3 in each set show biological triplicates, with column four representing a negative control. (**a**) M.m, 8% [vol/vol] CH_4_ in air; (**b**) M.t, 8% [vol/vol] CH_4_ in air; (**c**) M.m, 4% [vol/vol] CH_4_ in air; (**d**) M.t, 4% [vol/vol] CH_4_ in air; (**e**) M.m, 2% [vol/vol] CH_4_ in air; and (**f**) M.t, 2% [vol/vol] CH_4_ in air.

Given that methane is both the carbon source and the energy source of our methanotrophic cultures, the partitioning of carbon between energy-producing processes and biosynthesis has been estimated by measuring the changes in biomass, methane, and carbon dioxide over the growth period (Fig. 1). The carbon dioxide concentrations are the sum of the measured gaseous and modeled aqueous (HCO^−^_3_ and CO^2−^_3_) species.

Figure 1 depicts changes in the carbon balance relative to the initial carbon content of each culture over the 7-day experimental period. Within each set of four columns, the first three represent biological replicates, and the fourth represents a negative control. E0*–*E7 are the time series of samples from the external cultures, and C0 and C1 are for the start and end of the calorimeter cultures, respectively. For Fig. 1a, b, d, and f, all external culture samples (E0*–*E7) were collected at approximately 24 h intervals. In Fig. 1c, samples E0–E5 were collected at approximately 12 h intervals, followed by samples E6 and E7 at 48 h intervals. In Fig. 1e, samples E0–E3 were collected at 12 h intervals; E4, E5, and E7 at 24 h intervals; and E6 after a 48 h interval. The exact sampling times relative to the start of the experiment are provided in Fig S1 and S2.

A reduction in total carbon is observed between initial states (E0 and C0) and subsequent measurements (E1*–*E7 and C1). In most cases, these decreases can be attributed to methodological artifacts, as comparable reductions are observed within negative controls (fourth column within each set). Indeed, some loss of gas, and thus carbon, is expected during sampling and pressure measurements. Instances where carbon losses exceed those in the negative control may be due to variation in mass losses, or alternatively, to the production of metabolites (96–99) that have not been measured. Given that these instances are in the minority, we proceed under the assumption that the unaccounted carbon is lost from the system.

For *M. methanica* S1, more carbon is shown to be contained within biomass and less in carbon dioxide at the end of the growth period when compared to *M. trichosporium* OB3b (Fig. 1). Comparing carbon within the biomass and carbon dioxide produced with that present in the consumed methane (Table 1) reveals that for *M. methanica* S1, 27%*–*35% of the available carbon is converted to biomass, and 51%*–*59% is oxidized to carbon dioxide. For *M. trichosporium* OB3b, these percentages are 14%*–*22% and 64%*–*71%, respectively. The ratio of %CO_2_ to %biomass (Table 1) reinforces that for all our cultures, under the growth conditions provided, more methane is oxidized to carbon dioxide than is converted to biomass, and for *M. trichosporium* OB3b, the amount oxidized can be more than four times the amount converted to biomass.

The methane converted to biomass by both *M. methanica* S1 (27%*–*35%) and *M. trichosporium* OB3b (14%*–*22%) is less than previously measured within pure culture in a chemostat (55% and 30%–50%, respectively) (100). In naturally occurring methanotrophic communities, the carbon conversion efficiency (CCE) is also higher than reported here, observed to be consistent at approximately 50% (101). Lower values have, however, been measured in specific cases; for example, in a mixed culture of type II methanotrophs, as little as 10% of the available carbon was converted to biomass (100), and during pure culture of *M. capsulatus* (type I methanotroph), the CCE was observed to be between 31% and 42%, depending on the availability of copper (84). Indeed, the partitioning of substrate carbon can be sensitive to the environment and thus growth conditions (82). Our results are consistent with the observation that type I methanotrophs partition a greater proportion of available carbon to biomass compared to type II methanotrophs under similar conditions (82, 83, 101).

### Thermal output

**Fig 2:**
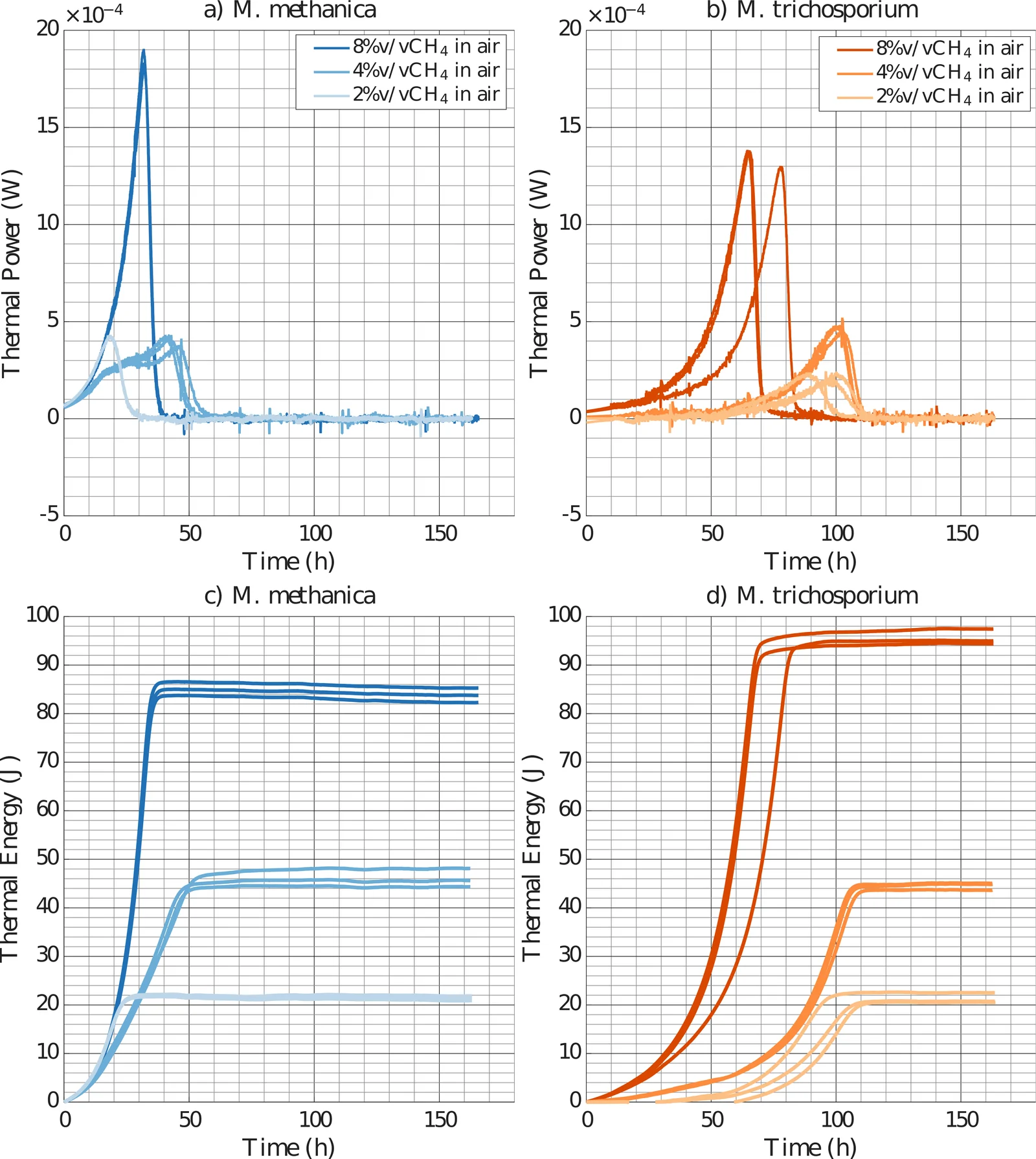
Thermal power and thermal energy dissipated during the batch growth of *M. methanica* S1 and *M. trichosporium* OB3b under three concentrations of methane (8%, 4%, and 2% [vol/vol] in air). Thermal power dissipation was measured directly using a Calmetrix I-CAL 4000 HPC Isothermal Calorimeter. Total thermal energy was determined via integration. (**a**) Thermal power dissipation from *M. methanica* S1, (**b**) thermal power dissipation from *M. trichosporium* OB3b, (**c**) total thermal energy dissipation from *M. methanica* S1, and (**d**) total thermal energy dissipation from *M. trichosporium* OB3b.

The power output of the calorimeter cultures is shown in Fig. 2a and b for *M. methanica* S1 and *M. trichosporium* OB3b, respectively, at the three methane concentrations (8, 4, and 2% [vol/vol] in air). In all cases, the power output increases exponentially to a peak and then drops off sharply at a time that coincides with the total consumption of methane within the external cultures (Fig. S1 and S2). This time point is also in line with the cessation of biomass and carbon dioxide production (Fig. S1 and S2). Given that


$$P=\frac{dQ}{dt},$$

where *P* is the instantaneous power output and *dQ*/*dt* is the rate of heat dissipation, integrating the power-time curves shown in Fig. 2a and b yields the cumulative heat released over the measurement period. These results are plotted in Fig. 2c and d for *M. methanica* S1 and *M. trichosporium* OB3b, respectively.

### The rate of heat dissipation

The maximum rate of heat dissipation (*P*_max_) exhibited by each species corresponds to the associated peak values within Fig. 2a and b. For *M. methanica* S1, these maximum rates are observed to be 1.849 ± 0.050 mW, 0.425 ± 0.011 mW, and 0.429 ± 0.003 mW for batch cultures grown with a concentration of 8%, 4%, and 2% (vol/vol) methane in air. For *M. trichosporium* OB3b, these rates are 1.356 ± 0.047 mW, 0506 ± 0.047 mW, and 0.242 ± 0.003 mW, respectively.

A mass-specific heat dissipation rate can be defined as the rate of heat dissipation normalized by the biomass present at a given time point. If this rate is constant, a linear relationship between biomass and thermal power dissipation is expected. This linear scaling has previously been observed in microorganisms (8). For *M. trichosporium* OB3b, grown under methane concentrations of 8%, 4%, and 2% vol/vol in air, we observe moderate linear relationships between biomass and heat production rates governed by equations $y=2.396x-3.958\times 10^{-4}$ ($R^{2}=0.75$), $y=0.903x-1.576\times 10^{-4}$ ($R^{2}=0.66$), and $y=1.007x-1.601\times 10^{-4}$ ($R^{2}=0.60$), respectively. Based on 95% confidence intervals, these correspond to mass-specific heat dissipation rates of 2.396±0.987, 0.903±0.411, and 1.007±0.521 W/g. Undertaking the same analysis for *M. methanica* S1, the mass-specific heat dissipation rates are determined to be 1.191±0.054, 0.524±0.152, and 1.432±0.390 W/g, respectively. The linear fits conducted for both *M. methanica* S1 and *M. trichosporium* OB3b are provided in Fig. S4.

While these rates substantially exceed those of larger organisms, they are not atypical for prokaryotes (102). Reported mass-specific heat production rates include 0.135*–*0.247 W/g for ruminal bacteria (103), up to 4.8 W/g for *E. coli* (104), and 13 W/g for the thermophilic methanogen *M. thermoautotrophicum* (11). It is important to note that for organisms utilizing a gaseous substrate, such as methanotrophs, the rate of heat production is related to the gas-liquid transfer rate (11). The absence of agitation in this study means that the reported mass-specific heat production rates underestimate those that would be observed in stirred cultures. Here, we point to the same reasons as explained previously in regard to the observed growth rates.

### Total heat dissipation and heat dissipation efficiencies

Despite the previously highlighted differences in the growth of *M. methanica* S1 and *M. trichosporium* OB3b (Table 1), the total heat dissipated is shown to be similar for both species. Our cultures of *M. methanica* S1 are shown to have dissipated 84.43±1.43, 45.92±1.79, and 21.66±0.39 J of heat when grown using concentrations of 8%, 4%, and 2% (vol/vol) methane in air, respectively. The corresponding values for *M. trichosporium* OB3b are 95.39±1.51, 44.46±0.70, and 21.41±0.92 J.

The quantity of heat dissipation (*Q*_dis_) enables the calculation of each species’ heat-production efficiency. This is achieved by relating the measured heat dissipation to the theoretical maximum heat obtainable from complete oxidation of the total supplied methane (*Q*_max_). *Q*_max_ is determined based on the stoichiometry of aerobic methane oxidation, which can be broken into two steps (74):


$${CH}_{4\left(g\right)}+O_{2\left(g\right)}\to {CH}_{2}O_{\left(g\right)}+H_{2}O_{\left(L\right)}$$

$${CH}_{2}O_{\left(g\right)}+O_{2\left(g\right)}\to {CO}_{2\left(g\right)}+H_{2}O_{\left(L\right)}$$

*Q*_max_ is calculated by multiplying the number of moles of methane consumed by the enthalpy of the complete reaction (−890.71 kJ/mol) (26). The corresponding efficiency (η_max_) is provided in Table 2.

**TABLE 2:** Thermal parameters for calorimeter (*cal*) batch cultures of *M. methanica* S1 and *M. trichosporium* OB3b grown under three concentrations of methane (8%, 4%, and 2% [vol/vol] in air)^*a*^

Species	Exp		*Q*_dis_ (J)		*Y_Q_*_/*X*_ (kJ/g)		*Y_Q_*_/*X*_ (kJ/Cmol)		*Y_Q_*_/*S*_ (kJ/mol)		*Y_Q_*_/*O*_ (kJ/mol)		η_max_ (%)	
	Cult	Methane												
			Mean	SD	Mean	SD	Mean	SD	Mean	SD	Mean	SD	Mean	SD
*M. m*	Cal	8%	84.43	1.43	59.46	0.21	1,519.38	5.35	508.21	1.75	373.96	16.20	57.06	0.20
		4%	45.92	1.79	78.27	4.19	2,000.09	107.02	543.38	19.91	416.60	17.10	61.00	2.24
		2%	21.66	0.39	57.66	1.94	1,473.42	49.57	516.12	12.99	426.33	31.45	57.94	1.46
*M. t*	Cal	8%	95.39	1.51	145.66	8.43	3,722.25	215.39	572.61	11.68	406.09	6.62	64.29	1.31
		4%	44.46	0.70	145.89	1.94	3,728.23	49.45	516.56	7.48	378.55	4.90	57.99	0.84
		2%	21.41	0.92	103.21	11.89	2,637.50	303.92	501.53	20.45	363.04	18.82	56.31	2.30

^*a*^ Reported values include total dissipated heat (*Q*_dis_), yields of dissipated heat per biomass produced (*Y_Q/X_*), per substrate consumed (*Y*_*Q*/*S*_), per oxygen consumed (*Y_Q/O_*), and the efficiency relative to the theoretical maximum heat stored in the substrate (η_max_). Means and standard deviations are shown from triplicates.

Both *M. methanica* S1 and *M. trichosporium* OB3b dissipate the majority of the maximum possible energy yield from the consumed substrate as heat, with η_max_ calculated between 57% and 61% for *M. methanica* S1 and between 56% and 64% for *M. trichosporium* OB3b. This proportion of heat evolved is comparable to that in the literature, where it is often stated that approximately 40%*–*60% of the energy available within the substrate is dissipated as heat (105–109).

### The heat yield and oxygen consumption

**Fig 3:**
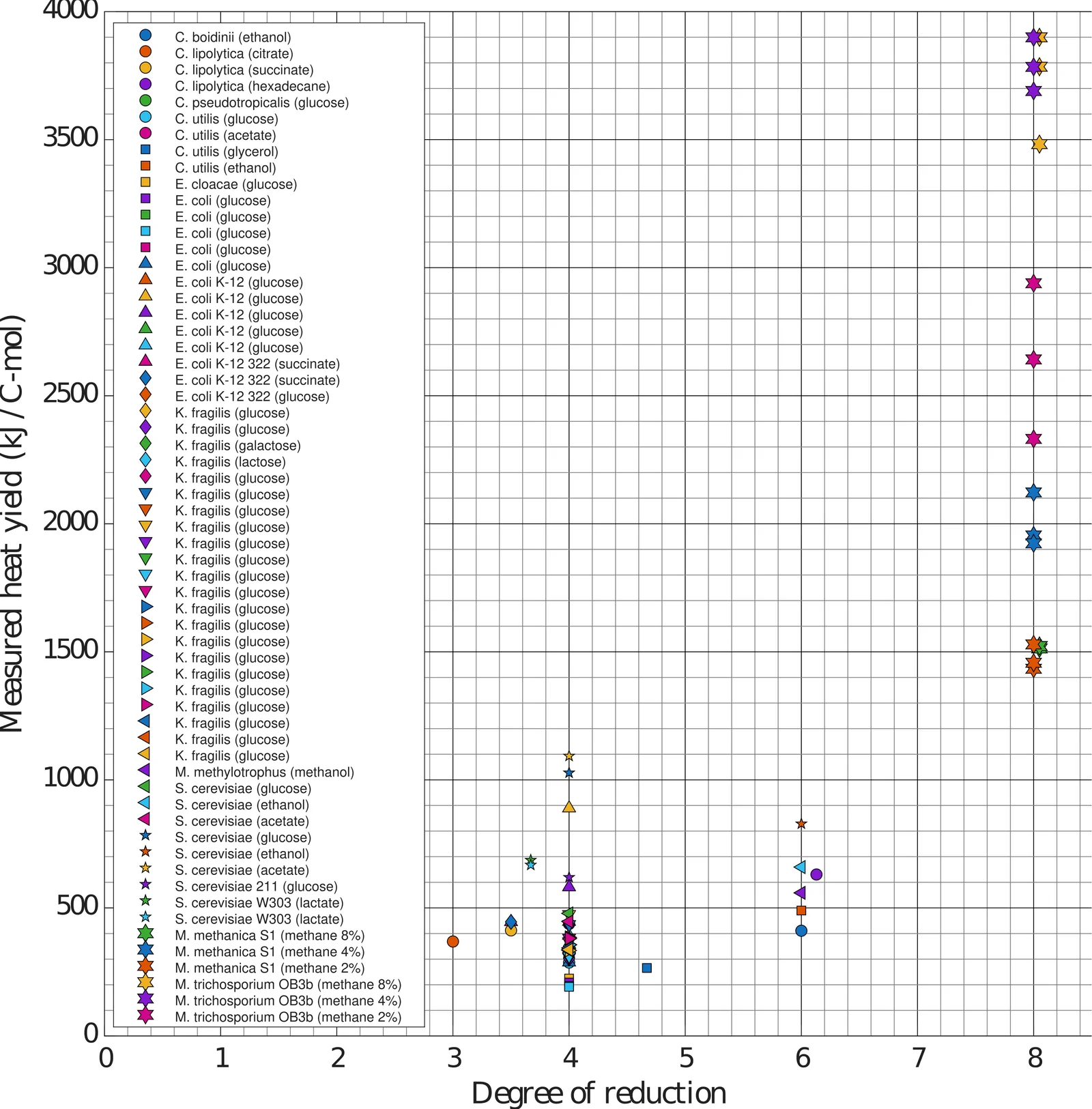
Comparison of experimentally measured heat yields from aerobic microorganisms against the degree of reduction of their associated substrates. The data set used was adapted from Cossetto et al. (25). Data points attributed to *M. methanica* S1 (methane 8%) and *M. trichosporium* OB3b (methane 8%) are given an *x* offset of +0.05 to improve visibility.

**Fig 4:**
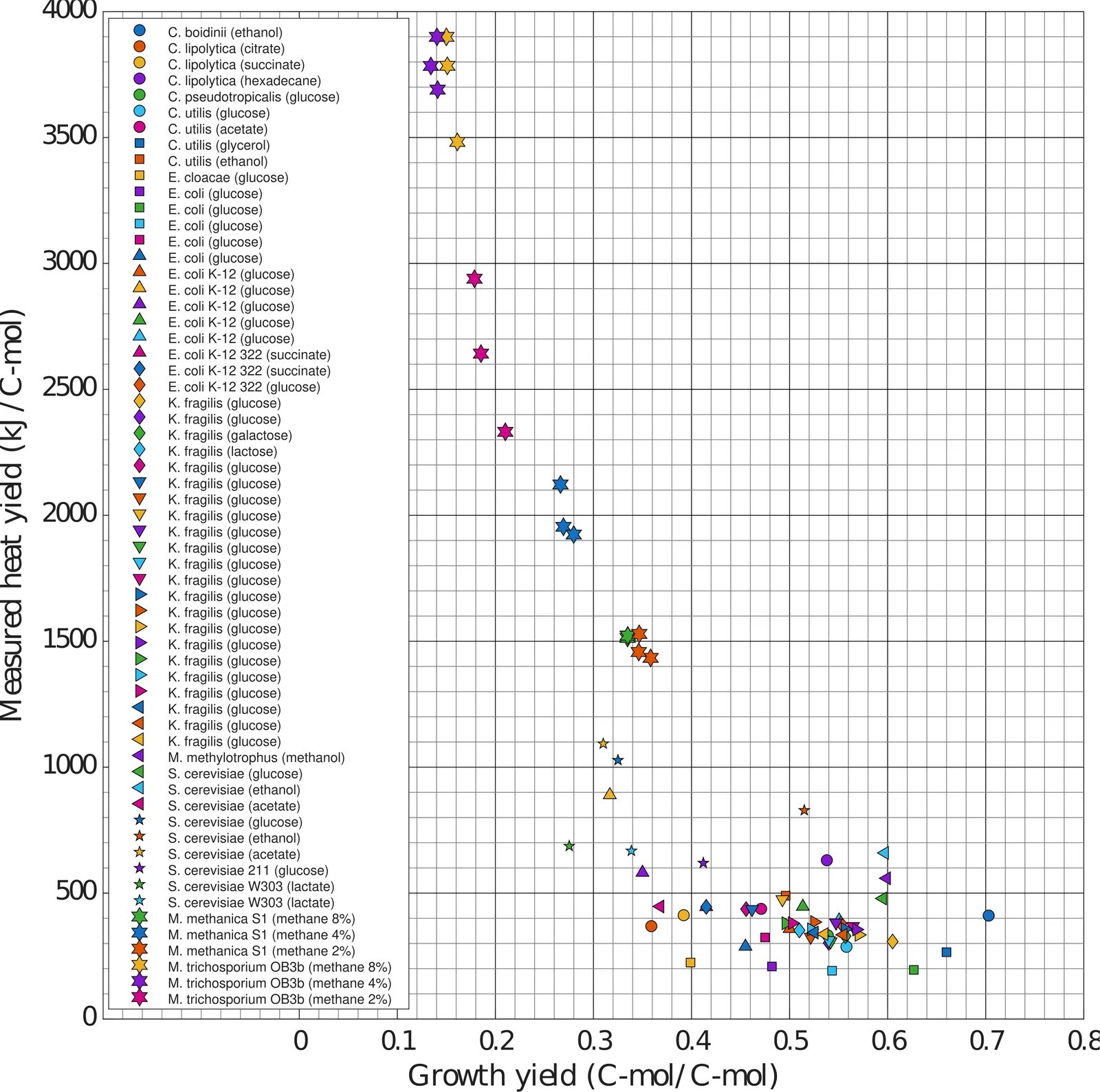
Comparison of experimentally measured heat yields from aerobic microorganisms against measured growth yields. The data set used was adapted from Cossetto et al. (25).

The heat dissipated per biomass produced ($Y_{Q/X}$) for *M. methanica* S1 and *M. trichosporium* OB3b are calculated between 1,473 and 2,000 kJ/Cmol and 2,638 and 3,728 kJ/Cmol, respectively, as shown in Table 2. These heat yields are compared with experimentally measured heat yields of other aerobic microorganisms in Fig. 3 and 4. These figures demonstrate that the values measured here are higher than those compared to and that *M. methanica* S1 and *M. trichosporium* OB3b are unique in the quantity of heat dissipated per biomass produced. While differences in the heat yields of the species provided in Fig. 3 and 4 can be attributed, at least in part, to the degree of reduction of the substrate used, the observed differences in $Y_{Q/X}$ between *M. methanica* S1 and *M. trichosporium* OB3b are independent of this and must therefore be the result of the aforementioned differences in growth. Indeed, Fig. 4 demonstrates the trade-off between heat yield and biomass yield for aerobic microorganisms—a relationship previously defined (3, 8). This suggests that changes in conditions that influence the internal partitioning of available carbon within methanotrophs may be used to optimize the associated heat yield. For methanotrophic bacteria, examples of these conditions may include the methane-oxygen concentration (36, 37, 94, 95) or the concentration of copper (84).

The yields of heat per oxygen consumed ($Y_{Q/O}$) are presented for our calorimeter cultures are presented in Table 2. In this study, oxygen consumption was estimated indirectly using an electron balance based on relevant degrees of reduction and the measurements of carbon dioxide production and biomass formation. The values obtained are therefore susceptible to uncertainties in these measurements and to the assumed biomass composition (${CH}_{1.8}O_{0.5}N_{0.2}$). This process also assumes no other metabolic products apart from biomass and carbon dioxide are formed, which is another source of uncertainty. $Y_{Q/O}$ is subsequently calculated from our measurements of heat dissipation. The values obtained within this study fall short of the theoretical value of 460 kJ/mol (3, 8, 110), but remain within the range of individual measurements presented previously (8, 19).

Adhering to the assumption that $Y_{Q/O}=460$ kJ/mol, the number of moles of oxygen can be determined directly from our measurements of *Q*_dis_. Indeed, the use of calorimetry as a tool for quantifying oxygen uptake within microorganisms has previously been suggested (111). This, in turn, facilitates the determination of yields, $Y_{O/S}$ and $Y_{O/X}$. For *M. methanica* S1, grown at methane concentrations of 8%, 4%, and 2% (vol/vol), $Y_{O/S}$ is calculated as 1.10±0.00, 1.18±0.04, and 1.12±0.28 mol/mol, respectively. $Y_{O/X}$ is calculated as 3.30±0.01, 4.35±0.23, and 3.20±0.11 mol/Cmol, respectively. For *M. trichosporium* OB3b, these values are 1.24±0.03, 1.12±0.02, and 1.09±0.04 mol/mol, and 8.09±0.47, 8.10±0.11, and 5.73±0.66 mol/C-mol, respectively.

Given that $Y_{Q/X}$ and thus $Y_{O/X}$ are lower for cultures of *M. methanica* S1 than *M. trichosporium* OB3b, a more efficient metabolism is suggested. We hypothesize that this difference arises from the distinct carbon assimilation pathways employed by type I and type II methanotrophs: the ribulose monophosphate (RuMP) cycle and the serine cycle, respectively. Indeed, thermodynamic analyses have demonstrated that the serine cycle incurs a greater energetic cost than the RuMP cycle due to its requirement of two ATP molecules (83, 112). Another hypothesis is that the observed behavior may reflect a partial decoupling of catabolic and anabolic processes in *M. trichosporium* OB3b under the imposed growth conditions. As previously stated, the presence of ambient carbon dioxide is known to inhibit the conversion of phosphoenolpyruvate to oxaloacetate within the serine cycle (87). Such inhibition may reduce the coupling between energy generation and carbon assimilation, leading to increased energetic losses. This uncoupling of catabolism from anabolism is known to impact heat dissipation (113) and, in our case, would disproportionately affect *M. trichosporium* OB3b, driving an increase in respective heat yields. Further studies directly linking pathway-level fluxes to heat production within methanotrophs would help disentangle the relative contributions of metabolic efficiency, regulatory decoupling, and environmental constraints.

While we observe no clear trend between the heat yields presented in Table 2 and the concentration of methane supplied, it is important to note that these concentrations exist within a similar physiological space. Increasing the methane concentration to levels at which oxygen becomes limiting may alter the heat yields owing to physiological changes associated with oxygen limitation (98). On the other hand, methane concentrations below 1,000 ppmv have been shown to alter the competitive balance between type I and type II methanotrophs, reversing trends observed at higher methane concentrations and demonstrating that type II methanotrophs exhibit a greater degree of oligotrophic adaptation under these conditions (114). Measuring heat yields at such methane concentrations would therefore provide insight into the energetic characteristics associated with ecologically relevant competitive regimes.

### Theoretical thermal energy recovery from wastewater using methanotrophic bacteria

To calculate the theoretical chemical energy present within wastewater based on COD, we must assume the form of organic compound present. Assuming the organic compound is methane, then the following can be written as:


$${COD+O}_{2}\to {CO}_{2}+H_{2}O$$

such that a single gram of COD equates to 1/32 mol O_2_, then, provided that the complete oxidation of methane requires 2 moles of O_2_, 1 mole of methane is equal to 64 g of COD. This, in turn, provides an energy content of wastewater of 13.9 kJ/g COD (115). We have demonstrated that *M. methanica* S1 and *M. trichosporium* OB3b can convert the chemical energy stored within methane to heat with an efficiency of 57%*–*61% and 56%*–*64%, respectively. Taking this into account, the maximum amount of heat each of these species can recover is 8.5 kJ/g COD and 8.9 kJ/g COD, respectively.

While this provides a preliminary theoretical estimate of the heat recoverable from wastewater using methanotrophic bacteria, the amount and rate of heat recovery achieved in practice will depend on the concentration and composition of influent chemical oxygen demand (COD). Indeed, the measured energy content of wastewater has been shown to vary between different waste streams (115, 116).

To establish the feasibility of harnessing metabolic heat from biological methane oxidation—for example, within wastewater treatment systems, practical constraints, and process parameters such as system scale, heat capture efficiency and retention, methane and oxygen loading rates, ambient temperature changes, pH, and biogas composition—will need to be addressed. Further research tasked with defining the relationships between these parameters and the rates and yields of metabolic heat from methanotrophs will be required. The findings presented in this study provide the quantitative foundation necessary for such further research and facilitate the refinement of existing thermodynamic models used to assess biological methane oxidation.

### Conclusions

In this study, we provide the first experimental quantification of metabolic heat dissipation in methanotrophic bacteria, demonstrating that a substantial fraction of the energy available from methane oxidation is released as heat under aerobic growth conditions. Across two model methanotrophs, *M. methanica* S1 and *M. trichosporium* OB3b, representing distinct carbon assimilation strategies, more than half of the maximum available energy was consistently dissipated (56%*–*64%), providing heat yields of up to 3,728 kJ/Cmol. These results highlight methane oxidation as an unusually heat-intensive microbial metabolism and confirm that methanotrophs are unique among aerobic microorganisms in the quantity of heat dissipated per biomass produced.

The differences in heat yield, oxygen consumption, and dissipation rates measured between *M. methanica* S1 and *M. trichosporium* OB3b coincided with associated growth behaviors. This observation suggests that intracellular carbon and energy partitioning, shaped by metabolic pathway choice and regulatory constraints, can be exploited to modulate heat dissipation. The observed species-specific behavior allows us to hypothesize that metabolic efficiency and partial decoupling between catabolic and anabolic processes may play a central role in governing microbial heat release.

Within an engineering context, the establishment of quantitative heat yields and heat production rates from methanotrophs provides key parameters for the design of environmental biotechnologies that incorporate biological methane oxidation. While we have briefly discussed the application of metabolic heat capture in relation to methane mitigation technologies, we believe the engineering impact to be much broader. Indeed, the use of methanotrophic bacteria to generate high-value products through industrial gas fermentation demonstrates another engineering application in which accurate knowledge of heat dissipation from methanotrophs would prove useful; specifically for informing the thermal management of such processes. Notwithstanding the practical engineering design considerations, the heat yields presented here provide quantitative insight relevant to bioenergy recovery and the broader thermodynamic optimization of engineered biological systems.

More fundamentally, it is our opinion that this work demonstrates the merit of undertaking precise physiological measurements and their relevance in tackling biochemical, ecological, and engineering questions. We encourage further studies that build upon these measurements to deepen our understanding of microbial energetics across diverse biological contexts. For example, comparing metabolic heat production under lower methane concentrations representative of the natural ecological niches occupied by different types of methanotrophs would help establish the role of metabolic heat dissipation in ecological competition and adaptation. Separately, investigating heat yields during growth on progressively more oxidized substrates, including methanol in place of methane, would provide further insight into the relationship between substrate oxidation state and metabolic heat production. Here, we also highlight the potential for synthetic biology to be applied to explore the consequences of metabolic decoupling and identify strategies for optimizing the metabolic heat yields of methanotrophic bacteria.

Ultimately, we hope that this work not only provides a quantitative basis for incorporating metabolic heat into the engineering of biotechnologies but also encourages broader experimental measurements of microbial bioenergetics, establishing metabolic heat dissipation as a quantitative physiological metric for characterizing microbial function across both natural and engineered environments.

## MATERIALS AND METHODS

### Growth conditions

#### Medium

All cultures of *M. methanica* S1 and *M. trichosporium* OB3b were grown using nitrate mineral salts (NMS) medium, as initially described by Whittenbury et al. (117). The exact recipe used can be found at https://store.ncimb.com/page/media_table (medium number 131).

#### Methane

Grade N2.5 (99.5%) methane was used as the carbon and energy substrate for all cultures. Methane was filtered using sterile Sartorius Minisart 0.2 µm PTFE syringe filters prior to the addition to cultures.

#### Stock cultures

Two species of methanotrophs were used throughout the experiments carried out: *M. methanica* S1 and *M. trichosporium* OB3b. Both strains were obtained from internal −80°C freezer stocks stored in the Advanced Research Center at the University of Glasgow. Stock cultures of both *M. methanica* S1 and *M. trichosporium* OB3b were maintained between subsequent experiments using a batch method. Cultures were grown at 30°C with no mixing in 3 *×* 250 mL serum bottles and an initial headspace concentration of 8% (vol/vol) methane in air. A 50:50 ratio between headspace and media was used. Both headspace and media were replaced on a weekly basis, where the amount of media replaced was tailored to ensure cultures were maintained at an OD_600_ of 0.100. Routine inspections using an EVOS microscope were undertaken to assess for possible contamination.

#### Calorimeter cultures

Batch cultures of *M. methanica* S1 and *M. trichosporium* OB3b were grown within a Calmetrix I-Cal 4000 HPC Isothermal Calorimeter at 30°C with no mixing. Bespoke 70 mL Simax borosilicate bottles were used for housing cultures (design specification provided in Fig. S5). To each bottle, 30 mL of inoculum was added, which was made up of 100 mL of stock culture (OD_600_ = 0.100) diluted to 1 L using NMS media. Gases were added through a series of injections using a 5 mL Hamilton gastight syringe model 1005 SL fitted with a sample lock and male luer RN hub (L) adapter; exact quantities of methane and air added to each culture are provided in Table S2, with the exact resulting headspace concentrations of methane provided in Tables S3 and S4. Biological triplicates were grown within three of the calorimeter’s sample chambers, while the fourth was used for a negative control. Prior to being placed inside the calorimeter, measurements of OD_600_, pH, pressure, and headspace composition were taken for each culture. Cultures were left within the calorimeter for a period of 7 days. On removal from the calorimeter, measurements of OD_600_, pH, pressure, and headspace composition were once again made.

#### External cultures

To produce time series, batch cultures of *M. methanica* S1 and *M. trichosporium* OB3b were also grown externally to the calorimeter. To ensure these cultures offered an accurate reflection of growth occurring within the calorimeter, they were conducted in parallel, with materials and growth conditions kept the same. Due to the volume required for the analytical analysis, sacrificial sampling was employed. Sets of 7 identical cultures were grown to facilitate daily sampling over the experimental period. Three of these sets were required to produce biological triplicates, and a further set was used as negative controls, providing a total of 28 cultures per experiment. Measurements of OD_600_, pH, pressure, and headspace composition were obtained at the beginning of the growth period and on the day of removal.

### Analytical methods

#### Optical density

The measurement of optical density was undertaken using a HACH DR1900 spectrophotometer. Measurements of absorption were made using a single wavelength of 600 nm. The HACH DR1900 was zeroed using NMS media. Each sample was aliquoted into 3 *×* 1 mL micro-cuvettes for measurement. The final value was determined by calculating the mean across all three measurements. All measurements of optical density were taken at room temperature (22°C).

#### pH

pH was measured using an Orion Star A215 pH Meter. Three-point calibration of the probe was undertaken before each set of measurements. Measurements of pH were taken at room temperature (22°C).

#### Cell dry weight

A calibration curve between optical density (OD_600_) and cell dry weight (CDW) was obtained to facilitate conversion between these two quantities. To measure CDW, stock cultures of *M. methanica* S1 and *M. trichosporium* OB3b were diluted to OD_600_ values of approximately 0.025, 0.050, 0.075, 0.100, 0.125, and 0.150. Samples were then vacuumed-filtered using 0.2-μm cellulose acetate membrane filters. Prior to use, the filters were dried at 105°C for 12 h, and the empty weight of each filter was measured. After filtration, the filters were once again dried at 105°C for 12 h before the final measurements of weight were recorded. Cell dry weight is determined by comparing the initial and final weights of each filter; these are then plotted against their respective optical density values. The resulting linear relationships are provided in Fig. S3. For samples with an OD_600_ of ≤0.100, 100 mL was filtered. For samples with an OD600 of >0.100, 50 mL was filtered.

#### Pressure

Measurements of pressure were taken using a Greisinger GDH 200 Manometer by attaching a 25G *×* 5/8" needle and 8 mm (outer diameter) PVC tubing to the positive port on the manometer. For each bottle, the readings were obtained on three separate occasions: first, after the addition of gases to determine the maximum pressure; second, after the first headspace sample was taken to record the initial pressure; and finally, a measurement was taken after the growth period had been completed to record the final pressure. In the first two instances, the pressure was measured at room temperature (22°C). For the final reading, pressure was measured at 30°C.

#### Headspace composition

Headspace composition was determined using gas chromatography. Headspace samples taken from cultures were stored within 3 mL DW Exetainer Vials and transported to the Scottish Universities Environmental Research Centre (SUERC) for analysis. Measurements of methane and carbon dioxide were made using a Hewlett Packard 5890 Gas Chromatograph with a thermal conductivity detector (TCD). Helium was used as the carrier gas, and a Hayesep-N polymer packed column was used with a column head pressure of 20 psi. The initial temperature was set at 50°C for 0.5 min, followed by a ramp rate of 40°C per min until a final temperature of 150°C. Readings were obtained from two 50 µL injections of each sample. Calibration and maintenance of the equipment were facilitated by SUERC.

#### Calorimetry

Measurements of thermal power were obtained from a Calmetrix I-Cal 4000 HPC Isothermal Calorimeter. The calorimeter was calibrated using Calmetrix calibration cement prior to use. A reference weight of 100 g was used within each reference chamber. Before each experiment, the calorimeter was set at the desired temperature for at least 24 h to stabilize, and an additional 24 h were used to produce baseline measurements. Measurements were taken over a period of 7 days every 60 s using the CalCommander software. To remove errors associated with the initial reduction in temperature, negative control measurements were used to smooth the associated settling time. The first 10 h of each data set were then removed and simulated by fitting a curve using subsequent data. To account for instrument offset, the mean of the final 12 h of each data set was subtracted from all data points. Further description of the data processing undertaken is provided in Supplemental material (Fig. S6 to S8).

### Calculations

#### Specific growth rates

Specific growth rates were estimated from the slope of a linear regression of log-transformed optical density values versus time. For each calculation, three consecutive optical density measurements were used. Identification of suitable points was undertaken through a sliding window analysis, which selected the interval that provided the best linear fit on the log-transformed growth curve. For *M. methanica* S1, this refers to the first three points measured, while for *M. trichosporium* OB3b, this refers to points 2*–*4.

#### Growth yields

Growth yields were calculated using the relationship


$$Y_{X/S}=\frac{\Delta X}{\Delta S},$$

where ΔX is the change in the mass of cell dry weight over the growth period and ΔS is the change in mass of methane over the same period.

#### Carbon partitioning percentages

Carbon partitioning was calculated by comparing the number of moles of carbon within biomass, carbon dioxide, and methane. The number of moles of carbon in biomass was calculated by assuming carbon makes up 47% of dry weight (84), and the molar weight of carbon is 12.0107 g/mol.

The number of moles of carbon within methane was calculated by first determining the total number of moles within the culture headspace using the ideal gas law. The number of moles of gaseous methane can then be determined from the measured concentration of methane. For dissolved methane, the number of moles is determined using Henry’s law, where Henry’s constant is adjusted for the correct temperature.

The number of moles of carbon dioxide was calculated in much the same way as for methane; however, contributions from the aqueous species HCO^−^_3_ and CO^2−^_3_ are also accounted for. Relevant constants are once again adjusted for temperature, pressure, and pH as required.

The percentages supplied in Fig. 1 are thus calculated as follows:


$${\%}_{{CH}_{4}}=\frac{\eta_{{CH}_{4}}}{\eta_{C,total,initial}}\times 100,$$

$${\%}_{{CO}_{2}}=\frac{\eta_{{CO}_{2}}}{\eta_{C,total,initial}}\times 100,$$

$${\%}_{biomass}=\frac{\eta_{C,biomass}}{\eta_{C,total,initial}}\times 100,$$

where $\eta_{{CH}_{4}}$ is the total number of moles of methane, $\eta_{{CO}_{2}}$ is the total number of moles of carbon dioxide (including HCO${}_{3}^{-}$ and CO${}_{3}^{2-}$), $\eta_{C,biomass}$ is the total number of moles of carbon in the biomass, and $\eta_{C,total,initial}$ is the total number of moles of carbon within all constituents at time zero.

The percentages provided in Table 1 are calculated slightly differently as follows:


$${\%}_{{CO}_{2}}=\frac{\eta_{{CO}_{2}}}{\eta_{CH_{4},consumed}}\times 100,$$

$${\%}_{biomass}=\frac{\eta_{C,biomass}}{\eta_{CH_{4},consumed}}\times 100,$$

where $\eta_{CH_{4},consumed}$ is the number of moles of CH_4_ consumed.

#### Thermal energy

Interval-wise energy contributions are determined through trapezoidal numerical integration of the thermal power measurements, such that


$$E_{i}=\int_{t_{i}}^{t_{i+1}}P(t)dt$$

The cumulative energy dissipated at each time point is thus determined through addition of the interval-wise energy contributions following:


$$E_{k}^{total}=\sum_{i}^{k}E(i)$$

where $E_{i}$ is the energy dissipated over the interval bounded by consecutive sampling times $t_{i}$ and $t_{i}+1$, P(t) is the measured thermal power as a function of time, $t_{i}$ denotes the $i_{th}$ sampling time, k is the index corresponding to the time point at which the cumulative energy is evaluated, and $E_{k}^{total}$ represents the total energy dissipated from the start of the experiment up to time $t_{k}$.

#### Thermal efficiency

The thermal efficiency ($\eta_{max}$) is calculated as


$$\eta_{max}=\frac{Q_{dis}}{Q_{max}}\times 100,$$

where *Q*_dis_ is the total heat dissipated, and *Q*_max_ is the maximum heat available based on the provided methane. *Q*_dis_ is calculated as the mean of the total energy dissipated from the point at which the thermal power dissipated returns to zero.

#### Thermal yields

Thermal yields were calculated using the relationships:


$$Y_{Q/X}=\frac{\Delta Q}{\Delta X},$$

$$Y_{Q/S}=\frac{\Delta Q}{\Delta S},$$

where ΔQ is the change in heat dissipation over the growth period, ΔX is the change in biomass over the growth period, and ΔS is the change in moles of CH_4_ over the growth period. For the calculation of $Y_{Q/O}$, the moles of oxygen consumed were first determined from carbon dioxide production and biomass formation. Based on changes in the oxidation state, each mole of CO_2_ corresponds to an eight electron transfer. Biomass, assumed to have the composition CH_1.8_O_0.5_N_0.2_, was assigned a degree of reduction of 4.2. Corrections were then applied to account for the electrons required to reduce the nitrogen source (KNO_3_). The remaining electron equivalents were attributed to oxygen reduction and converted to moles of O_2_ using four electrons per mole of O_2_. $Y_{Q/O}$ was then calculated analogously to other thermal yields.

## SUPPLEMENTAL MATERIAL

Supplemental material mbio.00980-26-s0001.pdf Supporting text, Fig. S1 to S8, and Tables S1 to S4.

ASM does not own the copyrights to Supplemental Material that may be linked to, or accessed through, an article. The authors have granted ASM a non-exclusive, world-wide license to publish the Supplemental Material files. Please contact the corresponding author directly for reuse.
