# Supplementary material for: Quantifying the metabolic heat from aerobic methanotrophs
Source: mBio. 2026 Sep 18;17(10):e00980-26. doi: 10.1128/mbio.00980-26 (PMC13712851; doi:10.1128/mbio.00980-26)
Supplement: Supplementary file 1 [file mbio.00980-26-s0001.pdf]

# Supporting Information for

## Quantifying the metabolic heat from aerobic methanotrophs

**Tymon A. Herzyk, Tania L. Gómez-Borraz, Rebeca Gonzalez-Cabaleiro and William T. Sloan**

**Tymon A. Herzyk**
**E-mail: Tymon.Herzyk@glasgow.ac.uk**

### This PDF file includes:

Supporting text
Figs. S1 to S8
Tables S1 to S4

## Supporting Information Text

## Results

**Time series.** Time series of the headspace concentration of methane and carbon dioxide, $OD_{600}$, system pressure and pH, for all external cultures of *M. methanica* S1 and *M. trichosporium* OB3b are provided in Figures S1 and S2 respectively. Results are provided for three distinct concentrations of methane (8%, 4% and 2% v/v in air). Final values obtained at the end of the growth period from the cultures within the calorimeter are also provided.

**$OD_{600}$ vs cell dry weight calibration curves.** A calibration curve was produced for converting between $OD_{600}$ and cell dry weight for *M. methanica* S1 and *M. trichosporium* OB3b separately, these are provided in Figure S3. Cultures were grown within 250 ml serum bottles at $30°C$ with no shaking and at methane concentration of $\leq 8\%$ v/v in air. Cultures of *M. methanica* S1 were diluted to $OD_{600}$ values of approximately 0.150, 0.125, 0.100, 0.075, 0.050 and 0.025. For *M. trichosporium* OB3b, dilution where made for $OD_{600}$ values of approximately 0.100, 0.075, 0.050 and 0.025. Triplicates of each dilution were vacuumed filtered using 0.2 $\mu$m cellulose acetate membrane filters. Prior to use, filters were dried at $105°C$ for 12 hours and the empty weight of each filter was measured. After filtration, filters were once again dried at $105°C$ for 12 hours before final measurements of weight were recorded. Cell dry weight is determined by comparing initial and final weights of each filter. For samples with an $OD_{600}$ of $> 0.100$, 50 ml was filtered. For samples with an $OD_{600}$ of $\leq 0.100$, 100 ml was filtered. For *M. methanica* S1, a relationship following $y = 0.3575x$ was observed to account for 83% of the variation ($R^2 = 0.8327$), thus $OD_{600} = 1.0 = 358$ mg/L ($CDW$). For *M. trichosporium* OB3b, a relationship following $y = 0.4967x$ was observed to account for 91% of the variation ($R^2 = 0.9065$), thus $OD_{600} = 1.0 = 497$ mg/L ($CDW$).

**Thermal power dissipation vs cell dry weight.** To determine the mass-specific rate of heat dissipation, the thermal power obtained from calorimeter cultures was plotted against cell dry weight measurements obtained from external cultures. Pairs between calorimeter cultures and those grown externally are made arbitrarily. Points are taken at the same time elapsed from when cultures were added into their respective growth environments. For calorimeter points that do not fall exactly on the same time point as those from external cultures, linear regression is used to determine the corresponding thermal power value. For determining linear fits, points during the growth phase are only considered.

## Materials

**Bespoke Calmetrix I-Cal 4000 HPC Isothermal Calorimeter bottles.** Bespoke bottles were designed for the Calmetrix I-Cal 4000 HPC Isothermal Calorimeter to facilitate the culture of methanotrophs within the system. Manufacture of bespoke bottles was undertaken by the University of Glasgow Glassblowing service. An engineering drawing of the initial bottle design is provided in Figure S5. Table S1 provides the measured weights and volumes of each bottle.

## Methods

**Addition of gases.** To make up the initial concentration of methane within each bottle a series of injections were made using a 5 mL Hamilton gastight syringe model 1005 SL fitted with a sample lock and male luer RN hub (L) adapter. A total of 19.01 ml of gas was added to each bottle through 4 separate injections. The exact composition of each injection for facilitating initial methane concentrations of 8%, 4% and 2% v/v in air are provided in Table S2. Tables S3 and S4 provide the measured initial headspace concentrations of methane from each bottle, for culture of *Methylomonas methanica* S1 and *Methylosinus trichosporium* OB3b respectively, when following the injection volumes provided in Table S2.

**Post-processing of calorimeter data.** The thermal power readings obtained from the Calmetrix I-CAL 4000 HPC Isothermal Calorimeter were processed through several steps to achieve removal of the baseline offset and settling period. Figure S6 depicts the raw measurements of thermal power from the Calmetrix I-CAL 4000 HPC Isothermal Calorimeter for each of the calorimeter experiments. As shown, each chamber within the calorimeter, relating to a single curve on each plot, exhibits an offset due to the materials used. To remove this offset, the last 12 hours of measurements (where no growth occurred) are average and this value was subtracted from all prior measurements; this results in the plots provided in Figure S7. Within Figures S6 and S7, a settling period is observed that affects the measurements made within the first 20 hours. To dampen the effect of the settling period, the first 20 hours of data from respective control samples are subtracted. The first 10 hours of each time series are then removed and a curve is fitted using data from the following 10 hours. For experiments undertaken at 4% and 2% v/v methane in air for *Methylomonas methanica* S1, only data from the following 5 hours are used to generate fits. The final result is the removal of the settling period as demonstrated by Figure S8.

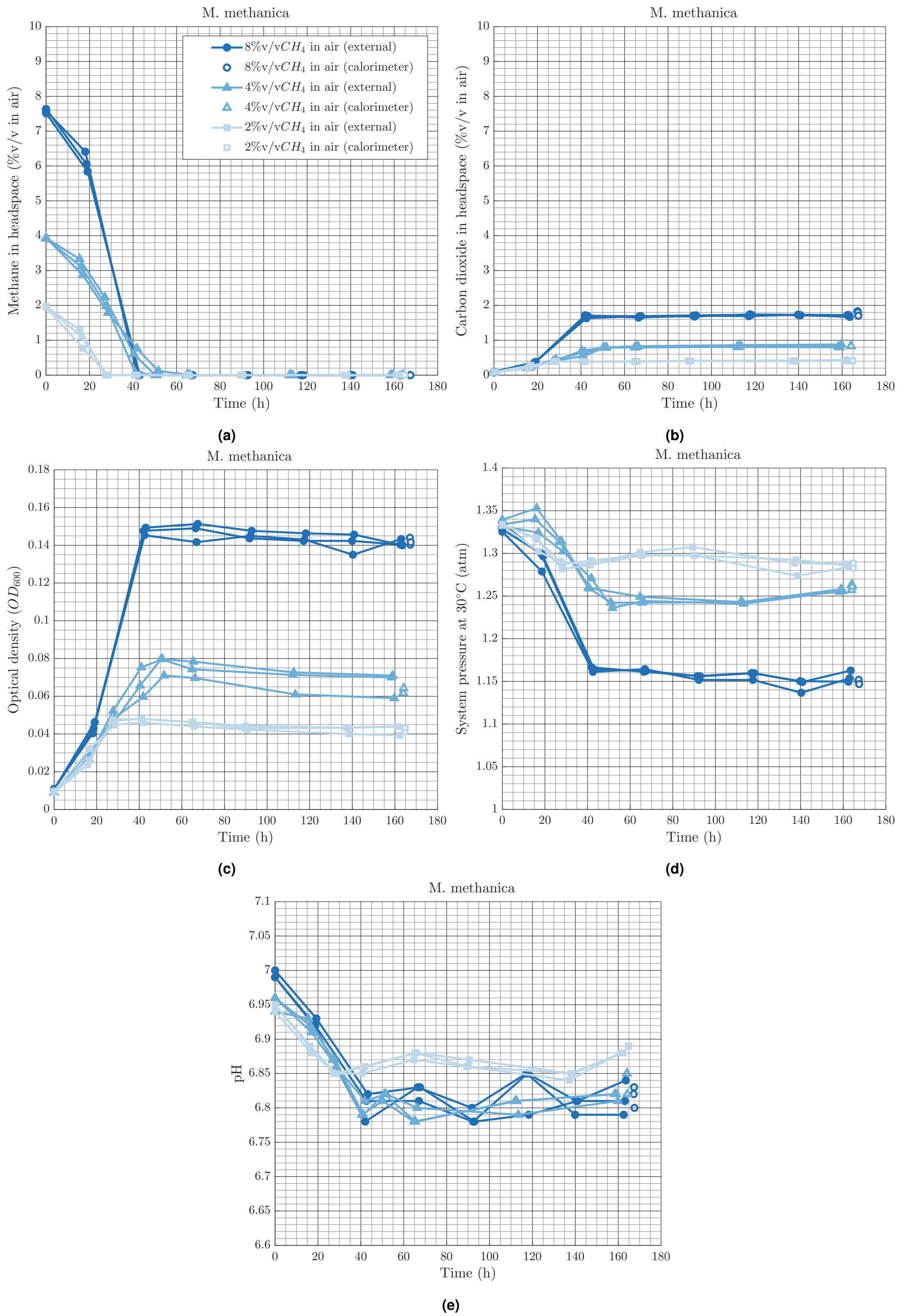


**Fig. S1.** Time series of growth parameters for external and calorimeter cultures of *Methylomonas methanica* S1, measured under three distinct concentration of methane (8%, 4% and 2% v/v in air) over a period of 7 days. a) Headspace concentration of methane, b) headspace concentration of carbon dioxide, c) optical density at 600 nm, d) system pressure, e) pH.

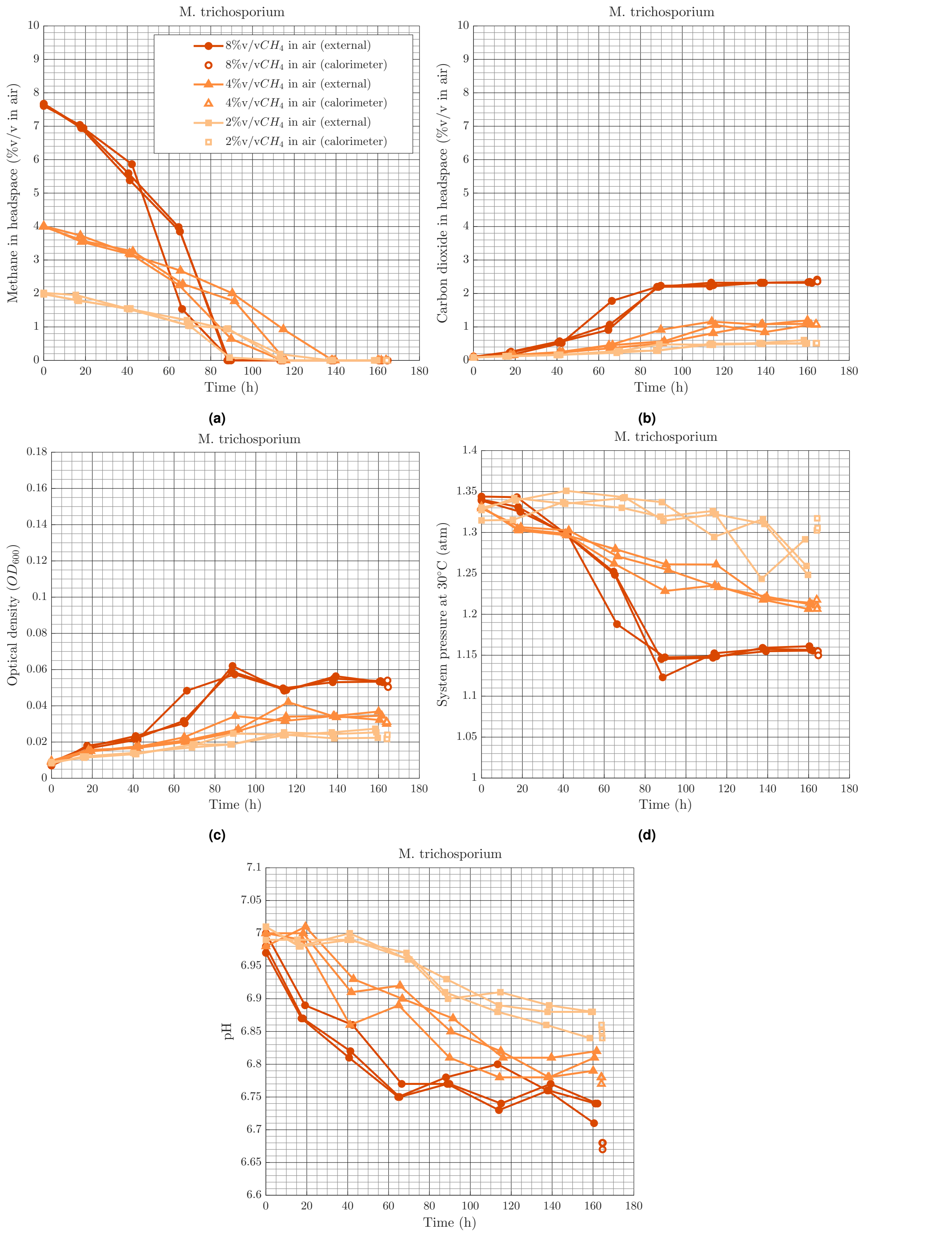


(a) (b) (c) (d) (e)

**Fig. S2.** Time series of growth parameters for external and calorimeter cultures of *Methylosinus trichosporium* OB3b, measured under three distinct concentration of methane (8%, 4% and 2% v/v in air) over a period of 7 days. a) Headspace concentration of methane, b) headspace concentration of carbon dioxide, c) optical density at 600 nm, d) system pressure, e) pH.

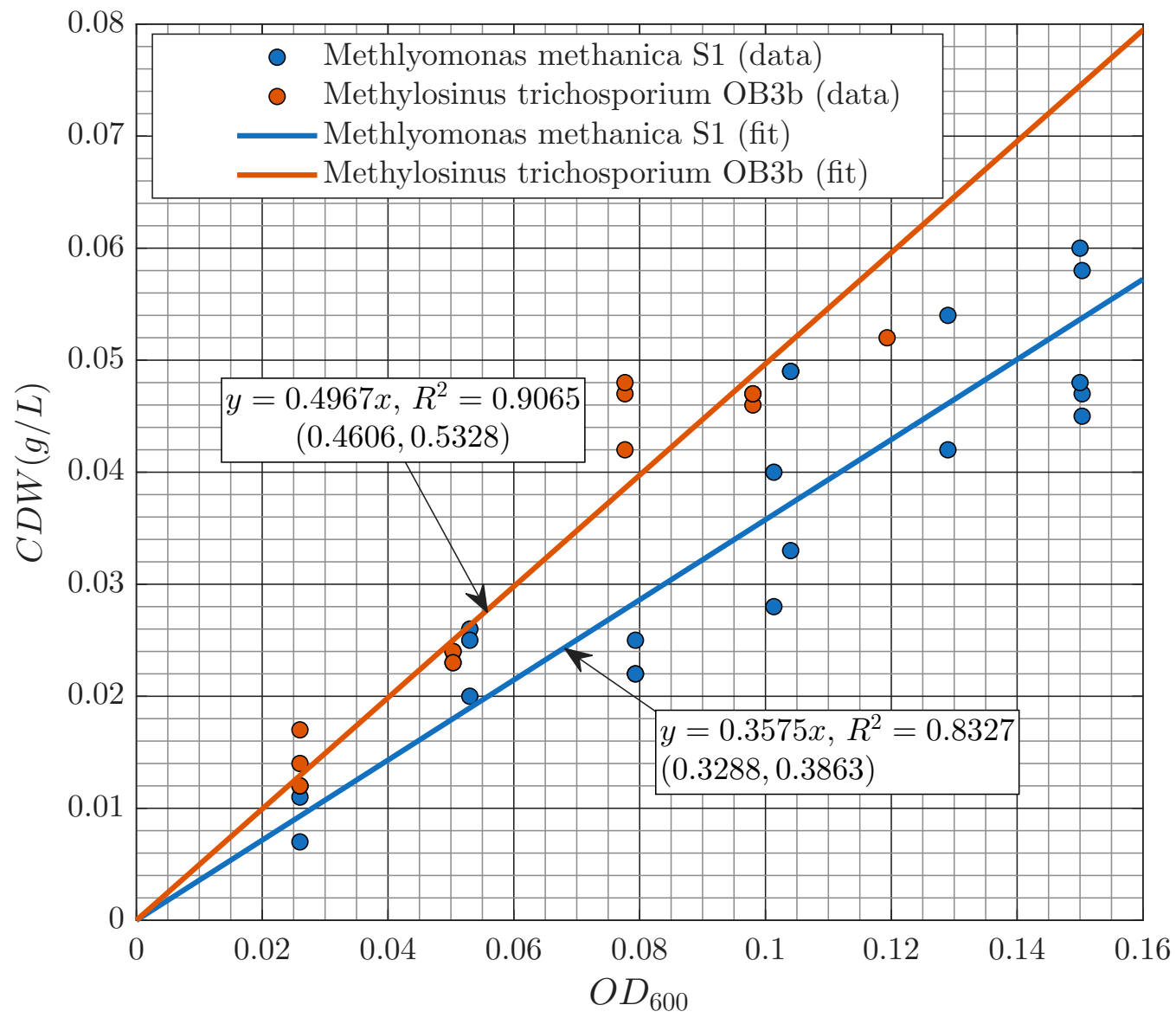


**Fig. S3.** Cell dry weight (CDW) plotted against optical density at 600 nm ($OD_{600}$) for *Methylomonas methanica* S1 and *Methylosinus trichosporium* OB3b. Linear fits are undertaken to provide formula for converting between measurements.

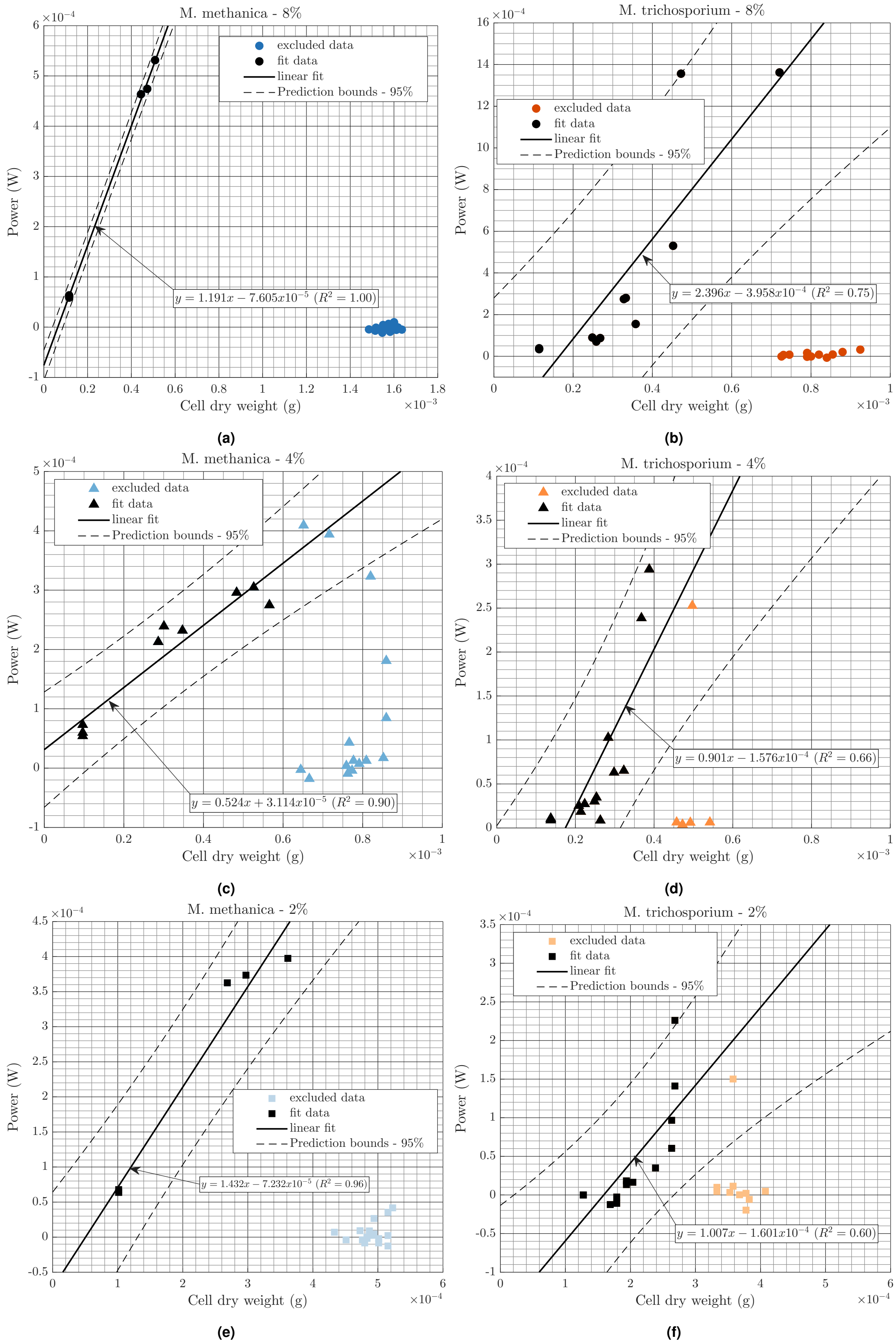


**Fig. S4.** Thermal power measurements plotted against cell dry weight measurments to determine mass-specific heat dissipation rates. Only data obtained during the growth phase are considered for determining linear fits a) *M. methanica* S1 $8\%$ v/v $CH_4$ in air, b) *M. trichosporium* OB3b $8\%$ v/v $CH_4$ in air, c) *M. methanica* S1 $4\%$ v/v $CH_4$ in air, d) *M. trichosporium* OB3b $4\%$ v/v $CH_4$ in air, e) *M. methanica* S1 $2\%$ v/v $CH_4$ in air, f) *M. trichosporium* OB3b $2\%$ v/v $CH_4$ in air.

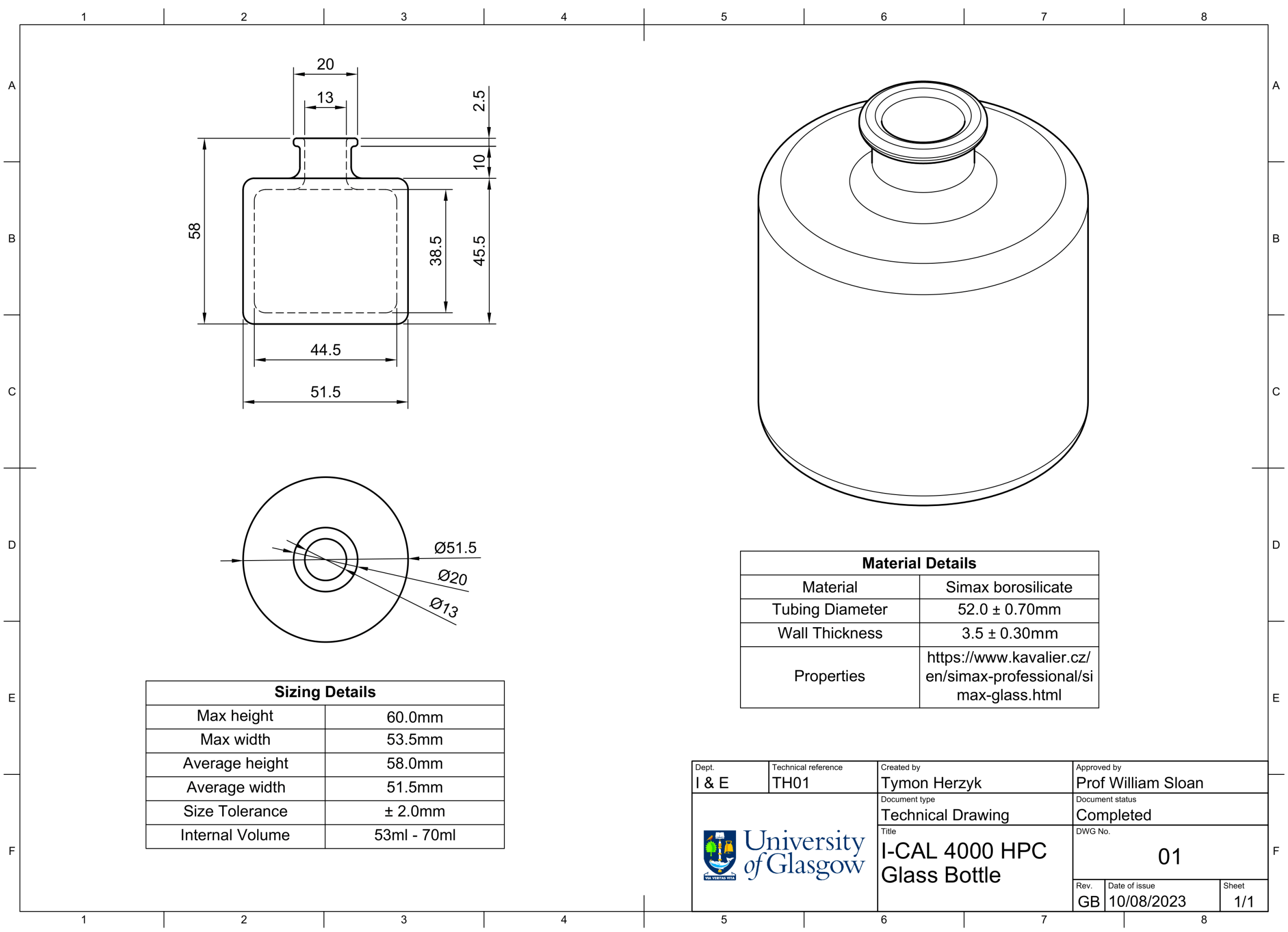


| Material Details | |
|---|---|
| Material | Simax borosilicate |
| Tubing Diameter | 52.0 ± 0.70mm |
| Wall Thickness | 3.5 ± 0.30mm |
| Properties | https://www.kavalier.cz/en/simax-professional/simax-glass.html |

| Sizing Details | |
|---|---|
| Max height | 60.0mm |
| Max width | 53.5mm |
| Average height | 58.0mm |
| Average width | 51.5mm |
| Size Tolerance | ± 2.0mm |
| Internal Volume | 53ml - 70ml |

| | | | |
|---|---|---|---|
| Dept. I & E | Technical reference TH01 | Created by Tymon Herzyk | Approved by Prof William Sloan |
| University of Glasgow | | Document type Technical Drawing | Document status Completed |
| | | Title I-CAL 4000 HPC Glass Bottle | DWG No. 01 |
| | | | Rev. GB · Date of issue 10/08/2023 · Sheet 1/1 |

**Fig. S5.** Initial engineering drawings created for bespoke Calmetrix I-CAL 4000 HPC Isothermal Calorimeter bottles. During final production wall thickness was decreases to 2.5mm. The internal neck diameter was also decreased during production from 13mm to 12mm.

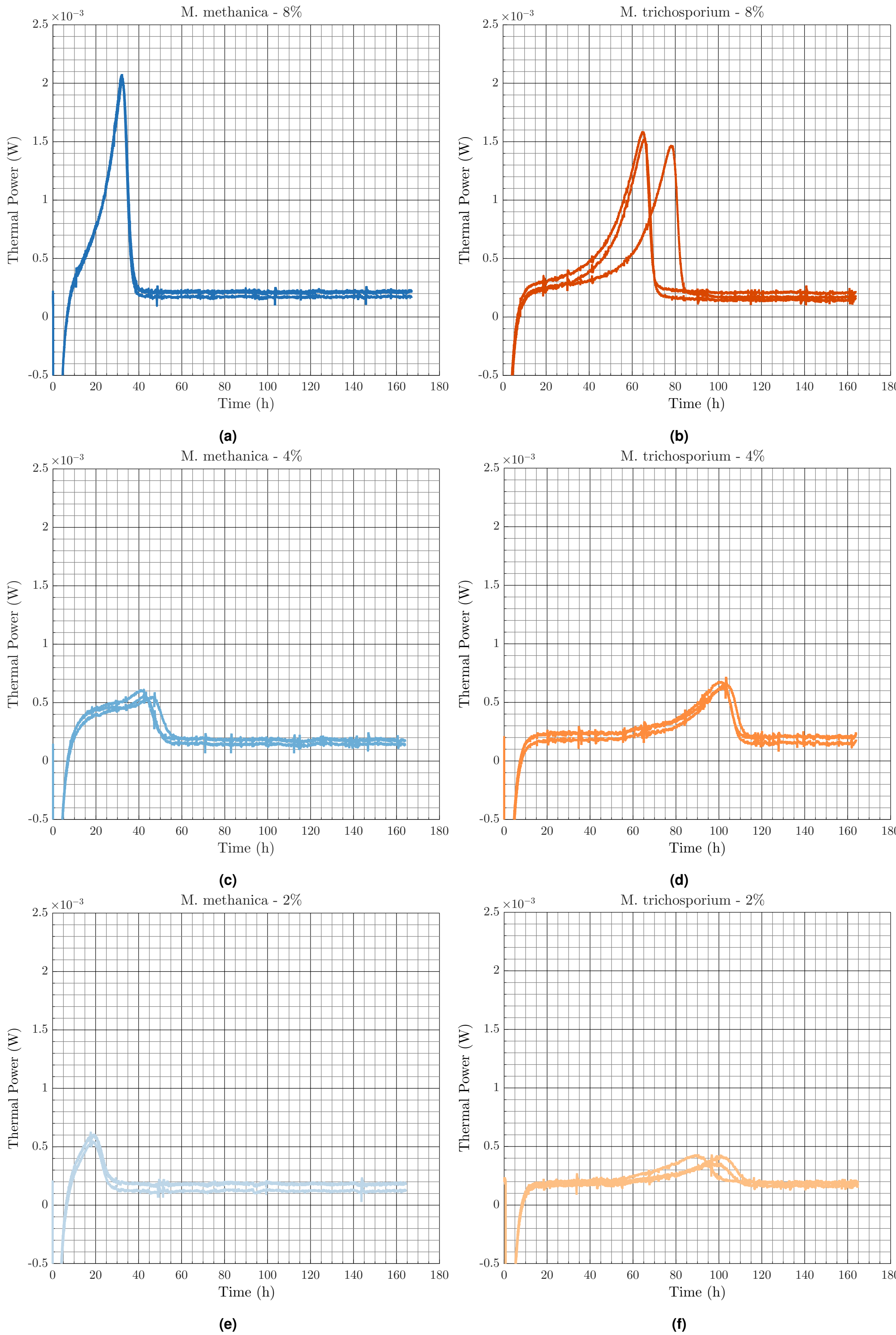


**Fig. S6.** Raw thermal power measurements made using a Calmetrix I-CAL 4000 HPC Isothermal Calorimeter from batch cultures of *M. methanica* S1 and *M. trichosporium* OB3b under three initial concentration of $CH_4$ ($8\%$, $4\%$ and $2\%$v/v in air). a) *M. methanica* S1 $8\%$ v/v $CH_4$ in air, b) *M. trichosporium* OB3b $8\%$ v/v $CH_4$ in air, c) *M. methanica* S1 $4\%$ v/v $CH_4$ in air, d) *M. trichosporium* OB3b $4\%$ v/v $CH_4$ in air, e) *M. methanica* S1 $2\%$ v/v $CH_4$ in air, f) *M. trichosporium* OB3b $2\%$ v/v $CH_4$ in air.

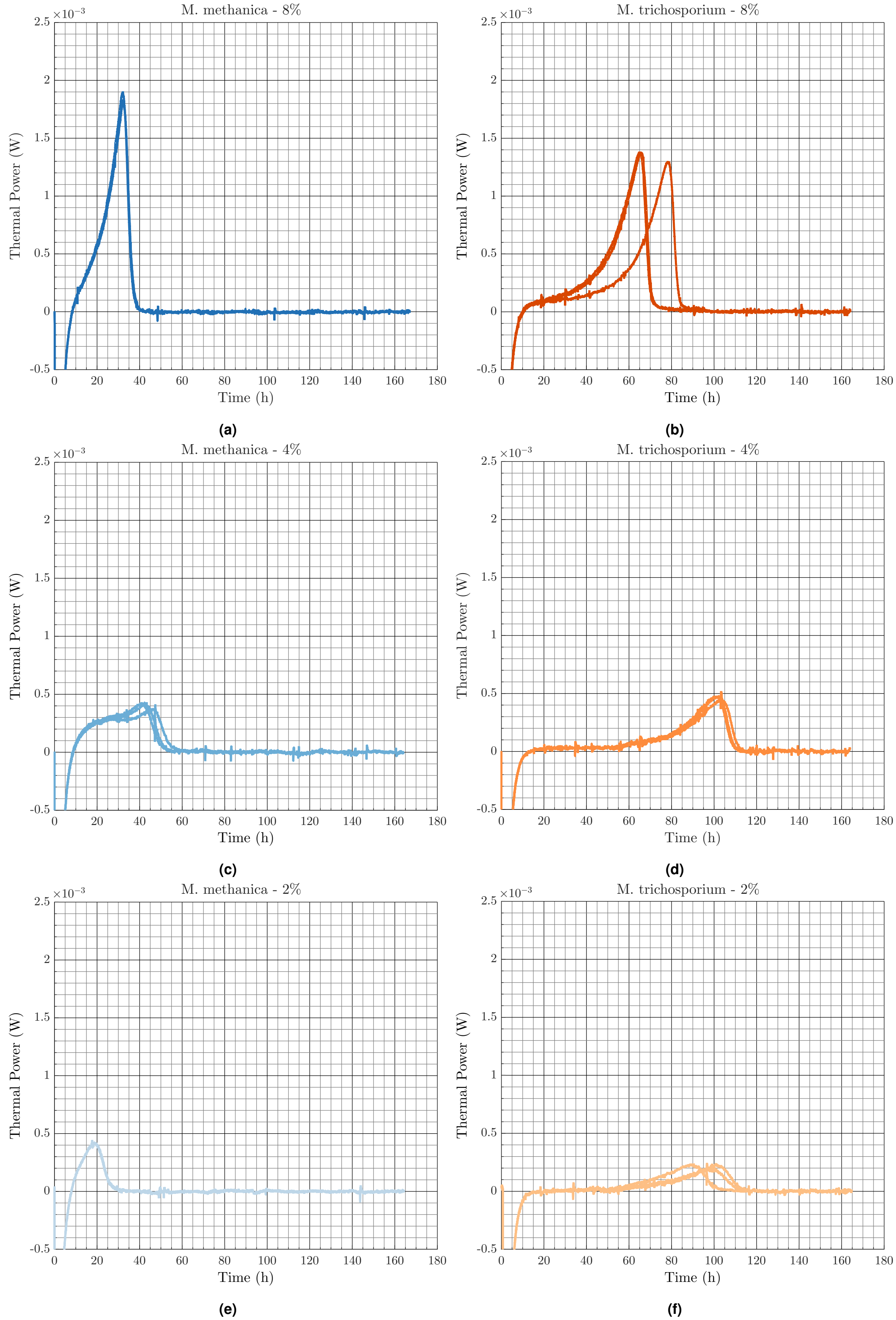


**Fig. S7.** Thermal power measurements provided in Figure S6 with baseline reduction. a) *M. methanica* S1 $8\%$ v/v $CH_4$ in air, b) *M. trichosporium* OB3b $8\%$ v/v $CH_4$ in air, c) *M. methanica* S1 $4\%$ v/v $CH_4$ in air, d) *M. trichosporium* OB3b $4\%$ v/v $CH_4$ in air, e) *M. methanica* S1 $2\%$ v/v $CH_4$ in air, f) *M. trichosporium* OB3b $2\%$ v/v $CH_4$ in air.

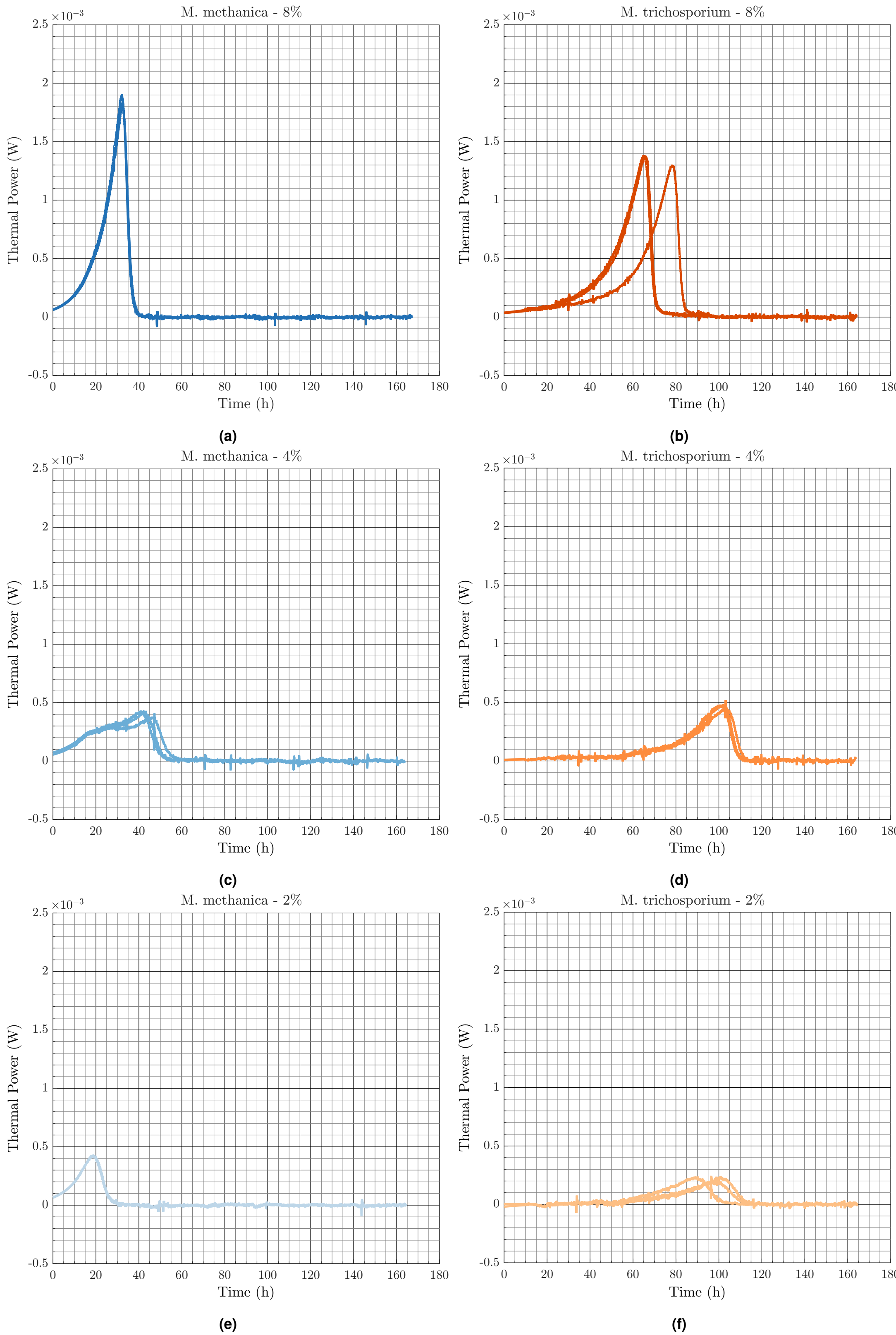


**Fig. S8.** Thermal power measurements provided in Figure S6 with baseline and settling period reduction. a) *M. methanica* S1 $8\%$ v/v $CH_4$ in air, b) *M. trichosporium* OB3b $8\%$ v/v $CH_4$ in air, c) *M. methanica* S1 $4\%$ v/v $CH_4$ in air, d) *M. trichosporium* OB3b $4\%$ v/v $CH_4$ in air, e) *M. methanica* S1 $2\%$ v/v $CH_4$ in air, f) *M. trichosporium* OB3b $2\%$ v/v $CH_4$ in air.

**Table S1. Weight and volume of bespoke Calmetrix I-CAL 4000 HPC Isothermal Calorimeter bottles.**

| name | number | empty weight $(g)$ | filled weight $(g)$ | volume $(ml)$ |
|---|---|---|---|---|
| CON 1 | 6 | 60.021 | 126.758 | 66.737 |
| CON 2 | 25 | 52.636 | 126.717 | 74.081 |
| CON 3 | 17 | 55.921 | 122.909 | 66.988 |
| CON 4 | 27 | 52.405 | 126.249 | 73.844 |
| CON 5 | 31 | 54.929 | 126.548 | 71.619 |
| CON 6 | 18 | 57.020 | 122.305 | 65.285 |
| CON 7 | 19 | 54.805 | 118.394 | 63.589 |
| SNG 1 | 3 | 53.804 | 122.107 | 68.303 |
| SNG 2 | 4 | 61.786 | 129.087 | 67.301 |
| SNG 3 | 5 | 61.128 | 129.839 | 68.711 |
| SNG 4 | 24 | 56.558 | 127.634 | 71.076 |
| SNG 5 | 7 | 59.307 | 128.237 | 68.930 |
| SNG 6 | 8 | 58.021 | 127.761 | 69.740 |
| SNG 7 | 10 | 60.118 | 128.922 | 68.804 |
| DUP 1 | 11 | 59.766 | 127.313 | 67.547 |
| DUP 2 | 26 | 51.552 | 121.18 | 69.628 |
| DUP 3 | 13 | 58.951 | 127.629 | 68.678 |
| DUP 4 | 14 | 53.335 | 124.173 | 70.838 |
| DUP 5 | 15 | 55.217 | 123.137 | 67.920 |
| DUP 6 | 16 | 54.462 | 121.978 | 67.516 |
| DUP 7 | 12 | 58.602 | 127.416 | 68.814 |
| TRI 1 | 20 | 57.101 | 123.81 | 66.709 |
| TRI 2 | 21 | 49.924 | 118.476 | 68.552 |
| TRI 3 | 22 | 50.737 | 121.47 | 70.733 |
| TRI 4 | 23 | 50.413 | 119.954 | 69.541 |
| TRI 5 | 28 | 50.407 | 118.457 | 68.050 |
| TRI 6 | 29 | 56.058 | 124.909 | 68.851 |
| TRI 7 | 32 | 56.604 | 125.37 | 68.766 |
| CAL 1 | 1 | 58.419 | 127.076 | 68.657 |
| CAL 2 | 2 | 57.808 | 126.212 | 68.404 |
| CAL 3 | 9 | 55.866 | 125.724 | 69.858 |
| CAL 4 | 30 | 58.029 | 126.548 | 68.519 |

**Table S2. Injection volumes of methane and air used during inoculation to provide target methane concentrations of $8\%$, $4\%$, and $2\%$v/v in air. Volumes were determined through trail and error.**

| Target $CH_4$ concentration ($\%$v/v in air) | Injection 1 | | Injection 2 | | Injection 3 | | Injection 4 | | Total | |
|---|---|---|---|---|---|---|---|---|---|---|
| | $CH_4$ (ml) | Air (ml) | $CH_4$ (ml) | Air (ml) | $CH_4$ (ml) | Air (ml) | $CH_4$ (ml) | Air (ml) | $CH_4$ (ml) | Air (ml) |
| 8 | 4.10 | 0.00 | 0.00 | 5.00 | 0.00 | 5.00 | 0.00 | 5.00 | 4.10 | 15.00 |
| 4 | 2.05 | 2.05 | 0.00 | 5.00 | 0.00 | 5.00 | 0.00 | 5.00 | 2.05 | 17.05 |
| 2 | 1.00 | 3.10 | 0.00 | 5.00 | 0.00 | 5.00 | 0.00 | 5.00 | 1.00 | 18.10 |

**Table S3. Measured initial methane concentrations for all bottles used for culture of *Methylomonas methanica* S1 in relation to target gas mixtures. Gases were added following the injection volumes provided in Table S2.**

| name | number | target $CH_4$ concentration 8% (% v/v in air) | target $CH_4$ concentration 4% (% v/v in air) | target $CH_4$ concentration 2% (% v/v in air) |
|---|---|---|---|---|
| CON 1 | 6 | 7.976 | 4.111 | 2.020 |
| CON 2 | 25 | 7.083 | 3.669 | 1.833 |
| CON 3 | 17 | 7.969 | 4.126 | 2.047 |
| CON 4 | 27 | 7.224 | 3.313 | 1.808 |
| CON 5 | 31 | 7.411 | 3.778 | 1.883 |
| CON 6 | 18 | 8.228 | 4.240 | 2.098 |
| CON 7 | 19 | 8.472 | 4.400 | 2.172 |
| SNG 1 | 3 | 7.394 | 3.967 | 1.980 |
| SNG 2 | 4 | 7.729 | 4.060 | 2.000 |
| SNG 3 | 5 | 7.614 | 3.961 | 1.962 |
| SNG 4 | 24 | 7.386 | 3.806 | 1.878 |
| SNG 5 | 7 | 7.574 | 3.965 | 1.941 |
| SNG 6 | 8 | 7.474 | 3.898 | 1.964 |
| SNG 7 | 10 | 7.447 | 3.915 | 1.929 |
| DUP 1 | 11 | 7.666 | 3.969 | 1.969 |
| DUP 2 | 26 | 7.400 | 3.851 | 1.956 |
| DUP 3 | 13 | 7.691 | 3.950 | 1.947 |
| DUP 4 | 14 | 7.385 | 3.788 | 1.903 |
| DUP 5 | 15 | 7.844 | 4.037 | 2.035 |
| DUP 6 | 16 | 7.810 | 3.995 | 2.006 |
| DUP 7 | 12 | 7.660 | 3.823 | 1.983 |
| TRI 1 | 20 | 7.842 | 4.028 | 2.011 |
| TRI 2 | 21 | 7.590 | 3.975 | 1.974 |
| TRI 3 | 22 | 7.353 | 3.831 | 1.895 |
| TRI 4 | 23 | 7.494 | 3.868 | 1.961 |
| TRI 5 | 28 | 7.618 | 3.970 | 2.011 |
| TRI 6 | 29 | 7.622 | 3.914 | 1.951 |
| TRI 7 | 32 | 7.572 | 3.941 | 1.953 |
| CAL 1 | 1 | 7.879 | 3.936 | 1.942 |
| CAL 2 | 2 | 7.693 | 3.957 | 1.933 |
| CAL 3 | 9 | 7.609 | 3.848 | 1.995 |
| CAL 4 | 30 | 7.738 | 4.047 | 1.933 |

**Table S4. Measured initial methane concentrations for all bottles used for culture of *Methylosinus trichosporium* OB3b in relation to target gas mixtures. Gases were added following the injection volumes provided in Table S2.**

| name | number | target $CH_4$ concentration 8% (% v/v in air) | target $CH_4$ concentration 4% (% v/v in air) | target $CH_4$ concentration 2% (% v/v in air) |
|---|---|---|---|---|
| CON 1 | 6 | 7.784 | 4.159 | 2.060 |
| CON 2 | 25 | 6.645 | 3.714 | 1.793 |
| CON 3 | 17 | 7.924 | 4.147 | 2.035 |
| CON 4 | 27 | 6.452 | 3.574 | 1.836 |
| CON 5 | 31 | 7.369 | 3.843 | 1.869 |
| CON 6 | 18 | 8.314 | 4.281 | 2.183 |
| CON 7 | 19 | 8.536 | 4.410 | 2.208 |
| SNG 1 | 3 | 7.918 | 4.034 | 2.039 |
| SNG 2 | 4 | 7.915 | 4.138 | 2.039 |
| SNG 3 | 5 | 7.735 | 3.989 | 2.047 |
| SNG 4 | 24 | 7.329 | 3.879 | 1.938 |
| SNG 5 | 7 | 7.584 | 4.048 | 1.879 |
| SNG 6 | 8 | 7.519 | 3.910 | 1.950 |
| SNG 7 | 10 | 7.704 | 3.963 | 2.023 |
| DUP 1 | 11 | 7.899 | 4.114 | 1.962 |
| DUP 2 | 26 | 7.519 | 3.934 | 1.936 |
| DUP 3 | 13 | 7.586 | 4.020 | 1.994 |
| DUP 4 | 14 | 7.317 | 3.832 | 1.907 |
| DUP 5 | 15 | 7.853 | 4.090 | 2.026 |
| DUP 6 | 16 | 7.779 | 4.111 | 2.029 |
| DUP 7 | 12 | 7.649 | 4.019 | 1.962 |
| TRI 1 | 20 | 7.937 | 4.194 | 2.064 |
| TRI 2 | 21 | 7.698 | 4.035 | 1.980 |
| TRI 3 | 22 | 7.261 | 3.880 | 1.930 |
| TRI 4 | 23 | 7.462 | 3.938 | 2.020 |
| TRI 5 | 28 | 7.625 | 4.072 | 2.086 |
| TRI 6 | 29 | 7.626 | 3.962 | 1.982 |
| TRI 7 | 32 | 7.651 | 4.027 | 2.048 |
| CAL 1 | 1 | 7.815 | 4.032 | 1.988 |
| CAL 2 | 2 | 7.789 | 4.042 | 1.998 |
| CAL 3 | 9 | 7.579 | 3.963 | 1.958 |
| CAL 4 | 30 | 7.703 | 4.048 | 2.069 |
